# A regulatory sub-circuit downstream of Wnt signaling controls developmental transitions in neural crest formation

**DOI:** 10.1371/journal.pgen.1009296

**Published:** 2021-01-19

**Authors:** Ana Paula Azambuja, Marcos Simoes-Costa

**Affiliations:** Department of Molecular Biology and Genetics, Cornell University, Ithaca, New York, United States of America; University of California, San Francisco, UNITED STATES

## Abstract

The process of cell fate commitment involves sequential changes in the gene expression profiles of embryonic progenitors. This is exemplified in the development of the neural crest, a migratory stem cell population derived from the ectoderm of vertebrate embryos. During neural crest formation, cells transition through distinct transcriptional states in a stepwise manner. The mechanisms underpinning these shifts in cell identity are still poorly understood. Here we employ enhancer analysis to identify a genetic sub-circuit that controls developmental transitions in the nascent neural crest. This sub-circuit links Wnt target genes in an incoherent feedforward loop that controls the sequential activation of genes in the neural crest lineage. By examining the cis-regulatory apparatus of Wnt effector gene *AXUD1*, we found that multipotency factor SP5 directly promotes neural plate border identity, while inhibiting premature expression of specification genes. Our results highlight the importance of repressive interactions in the neural crest gene regulatory network and illustrate how genes activated by the same upstream signal become temporally segregated during progressive fate restriction.

## Author summary

The neural crest is a migratory stem cell population that plays a crucial role in the development of vertebrate embryos. These cells contribute to multiple tissues and organs, including the craniofacial skeleton, the peripheral nervous system, and the pigmentation of the skin. Neural crest formation requires sequential expression of sets of genes that gradually change the identity of progenitor cells. Here we characterize a gene circuit that plays an important role in these developmental transitions. We show that a pair of transcription factors downstream of the Wingless (Wnt) signaling pathway (*SP5* and *AXUD1*) control the temporal activation of neural crest genes. While SP5 drives the expression of genes expressed at gastrula stages, *AXUD1* promotes neural crest genes transcribed during neurulation. We found that these regulators are linked by inhibitory interactions that prevent overlap between transcriptional states. Our results shed light on how the architecture of gene regulatory circuits underlies the timing of developmental transitions during neural crest development.

## Introduction

The neural crest is a migratory stem cell population that gives rise to multiple components of the vertebrate body plan, including the peripheral nervous system and the craniofacial skeleton [1]. The formation of neural crest cells is controlled by a gene regulatory network (GRN) composed of signaling systems, transcription factors, and other regulatory proteins [2–4]. Hence, the process of neural crest formation can be subdivided into a series of distinct regulatory modules that flow logically from one to the next. First, extracellular signaling molecules (WNTs, FGFs, BMPs and Notch) present in the early embryo initiate the induction of the neural plate border. The sequential activation of these signal transduction pathways promotes the expression of transcription factors termed neural plate border specifier genes (e.g., *PAX7*, *MSX1*, *ZIC1*, *TFAP2A*) [5]. These factors cooperate with signaling systems to activate transcription of neural crest specifier genes, which include neural crest markers like *SNAI2*, *FOXD3*, and *SOX10*. Neural crest specifier genes, in turn, activate the effector genes that endow these cells with their unique properties, such as multipotency and migratory capabilities [4].

The neural crest GRN is one of the most comprehensive regulatory networks described for a vertebrate cell type, and has served as a platform for investigating molecular mechanisms involved in cell fate commitment and vertebrate evolution [6–8]. Yet, we still have a superficial understanding of the molecular logic encoded in the architecture of the network. Metazoan GRNs are structured as a collection of sub-circuits, in which small groups of genes are linked together by positive or negative interactions [9,10]. GRN sub-circuits are the functional units of the program, performing tasks required to change transcriptional states or to establish spatial domains of gene expression [11]. While past functional and biochemical studies resulted in a substantial expansion of the neural crest GRN, only a few examples of regulatory sub-circuits have been described in this cell type [12]. Delineation of additional regulatory motifs is necessary to understand how this genetic program orchestrates developmental transitions.

Recently, we have identified Wnt target gene *AXUD1* (also known as *CSRNP-1*) as a critical component of the neural crest GRN [13]. This transcription factor forms a protein complex with PAX7 and MSX1 to directly promote the expression of specifier genes. Notably, while PAX7 and MSX1 are present during gastrula stages[14,15], specifier genes including *FOXD3* and *SOXE* factors are only expressed following the start of *AXUD1* expression at Hamburger and Hamilton stage 7 (HH7) [13]. This indicates that AXUD1 plays an essential role in activating the neural crest specification program. However, direct activators of this transcription factor have yet to be identified. While *AXUD1* is hypothesized to be downstream of canonical Wnts, it does not accurately recapitulate the activity of this signaling system, as Wnt ligands are already present in the early gastrula [16], but *AXUD1* transcription only starts during neurulation. This discrepancy suggests a complex mode of regulation that may involve integration of numerous inputs. Identifying how upstream factors control *AXUD1* expression may reveal new mechanisms responsible for the emergence of neural crest identity.

Here we describe a regulatory sub-circuit that controls the onset of neural crest formation in vertebrate embryos. By surveying the *AXUD1* locus with chromatin conformation capture, we identified a tissue-specific enhancer that interacts with the promoter of this gene in neural crest cells. Dissection of this enhancer allowed for the delineation of a sub-circuit that operates downstream of Wnt signaling to control the temporal expression of neural crest genes. *AXUD1* is activated by Wnt signaling and neural plate border genes *TFAP2A*, *MSX1*, and *ZIC1*. Crucially, direct repression by Wnt effector gene *SP5* restricts the timing of *AXUD1* expression to prevent premature expression of specification genes. We assembled these interactions in a sub-circuit that links Wnt-target genes in an incoherent feedforward circuit, which controls the sequential activation of genes in the neural crest lineage. Our results highlight the importance of repression in the neural crest GRN and demonstrate how the interactions between Wnt target genes drive progressive fate restriction.

## Results

### Tissue-specific regulatory elements interact with the *AXUD1* promoter

**Fig 1:**
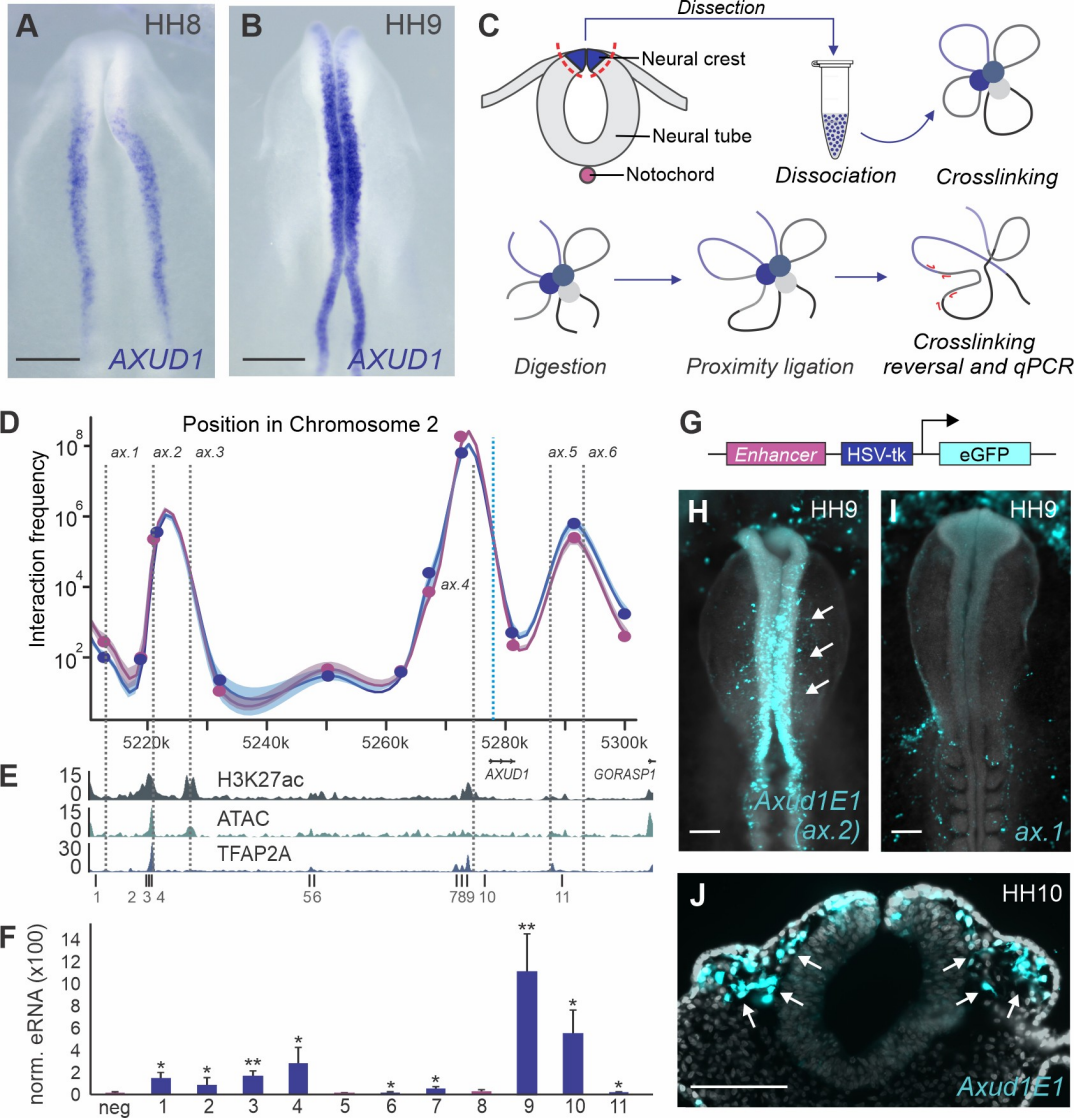
Chromatin conformation capture identifies a tissue-specific enhancer that interacts with the *AXUD1* promoter. **(A-B)** Whole mount *in situ* hybridization for *AXUD1*, depicting the specific mRNA expression during neural crest specification stages HH8 (**A**) and HH9 (**B**). **(C)** Schematic representation of a Chromosome Conformation Capture-qPCR (3C-qPCR) experiment. Crosslinked neural crest cells were incubated with restriction enzyme NCOI and a DNA ligase. These steps allow the formation of hybrid DNA molecules combining restriction fragments that were in close proximity in nuclei of the cells. Primers spanning the *AXUD1* locus were paired with a primer anchored in the *AXUD1* promoter to amplify hybrid DNA junctions and quantify the interaction frequency with the promoter. **(D-F)** Identification of active enhancers in the *AXUD1* locus. 3C-qPCR interaction map for the *AXUD1* locus reveals regions of high interaction frequency with the promoter region (blue dotted line, **D**). Gray dotted lines highlight the six elements tested in transient transgenesis assays (see below). Error bars represent ± SEM. Purple and blue lines in (**D**) represent two replicates of the same 3C experiment. ATAC-seq, H3K27ac and TFAP2A CUT&RUN profiles at *AXUD1* locus depict regions of accessibility and active chromatin regions (**E**). (**F**) eRNA quantification (RT-PCR, normalized to reference gene) for the regions numbered in (**E**) indicates the level of transcription in the promoter region and putative distal regulatory elements. Error bars represent ± SEM. Statistical significance determined via an unpaired t-test. **(G**) Reporter vector used in transgenesis reporter assays. The construct consists of the candidate enhancer region cloned upstream of the HSV-tk minimal promoter driving eGFP expression. **(H-J**) The *Axud1E1* element is active in neural crest cells. *In vivo* activity of *Axud1E1* as shown by eGFP expression in reporter assays (**H**). *Axud1E1* recapitulates endogenous gene expression in dorsal neural folds (arrows), while the putative enhancer *ax*.*1* displayed no specific activity (**I**). Transverse cryosection from a HH10 embryo electroporated with the *Axud1E1* enhancer illustrates eGFP expression in the dorsal neural tube and migratory neural crest cells (**J**). HH, Hamburger and Hamilton. Scale bars represent 500μm (**A-B**), 200μm (**H-I**) and 100μm (**J**). *p < 0.05, **p < 0.01.

*AXUD1* is first detected in the neural crest lineage at (HH7), before the onset of specification markers *FOXD3*, *ETS1* and *SOX9* [13]. *In situ* hybridization in avian embryos shows robust expression of this transcription factor at HH8-9 (Fig 1A and 1B). To identify the cis-regulatory elements that control *AXUD1* expression, we first employed Chromatin Conformation Capture (3C) in neural crest cells isolated from embryos at these developmental stages. Dorsal neural folds from the cranial region of HH8-9 embryos were micro-dissected with iridectomy scissors and dissociated into a single-cell suspension. Cells were fixed with formaldehyde, and the crosslinked chromatin was digested with restriction enzyme NCOI (Fig 1C). After proximity ligation, we used RT-PCR to estimate the frequency of interaction between the promoter and sectors of the *AXUD1* locus (defined by the presence of NCOI sites, see Methods). For this analysis, we profiled a region spanning ~100kb between the two genes that flank *AXUD1*. The resulting 3C plot (Fig 1D) contained the expected high interaction values near the promoter, but also uncovered a large interaction peak ~55kb upstream of the transcription start site (TSS). This interaction profile was consistent in all biological replicates of the experiment (n = 3).

To identify active enhancers of *AXUD1*, we characterized genomic regions that interact with its promoter using datasets of chromatin accessibility (ATAC-seq), H3K27Ac association and TFAP2A occupancy in neural crest cells [17]. TFAP2A is a pioneer transcription factor that marks active neural crest enhancers [18]. This analysis led to the identification of 11 putative cis-regulatory elements (Fig 1E). To test if these elements were active, we performed RT-PCR for enhancer RNA (eRNA) in dissected neural folds of HH9 chicken embryos. We detected high levels of transcription both in TFAP2A-occupied putative enhancers adjacent to the 3C peak (elements 1–4) and in elements adjacent to the *AXUD1* promoter (elements 9–10) (Fig 1F). To test if these regions were able to drive gene expression, we conducted transgenic reporter assays in chick embryos. Putative enhancers (*ax*.*1*-*ax*.*6*, Fig 1D and 1E) were cloned in the pTK-eGFP vector [19] (Fig 1G) and electroporated in the ectoderm of gastrula-stage embryos (HH4). Of these elements, only *ax*.*2* was active in neural crest cells at HH9 (Fig 1H), whereas the other constructs tested (S1 Table) were unable to drive reporter expression (Fig 1I). Activity of *ax*.*2* was consistent with previous testing of putative neural crest enhancers based on genome accessibility [20]. Histological analysis confirmed that ax.2 was specifically active in neural crest cells (Figs 1J and S1), and we thus refer to it as *AXUD1* enhancer 1 (*Axud1E1*).

**Fig 2:**
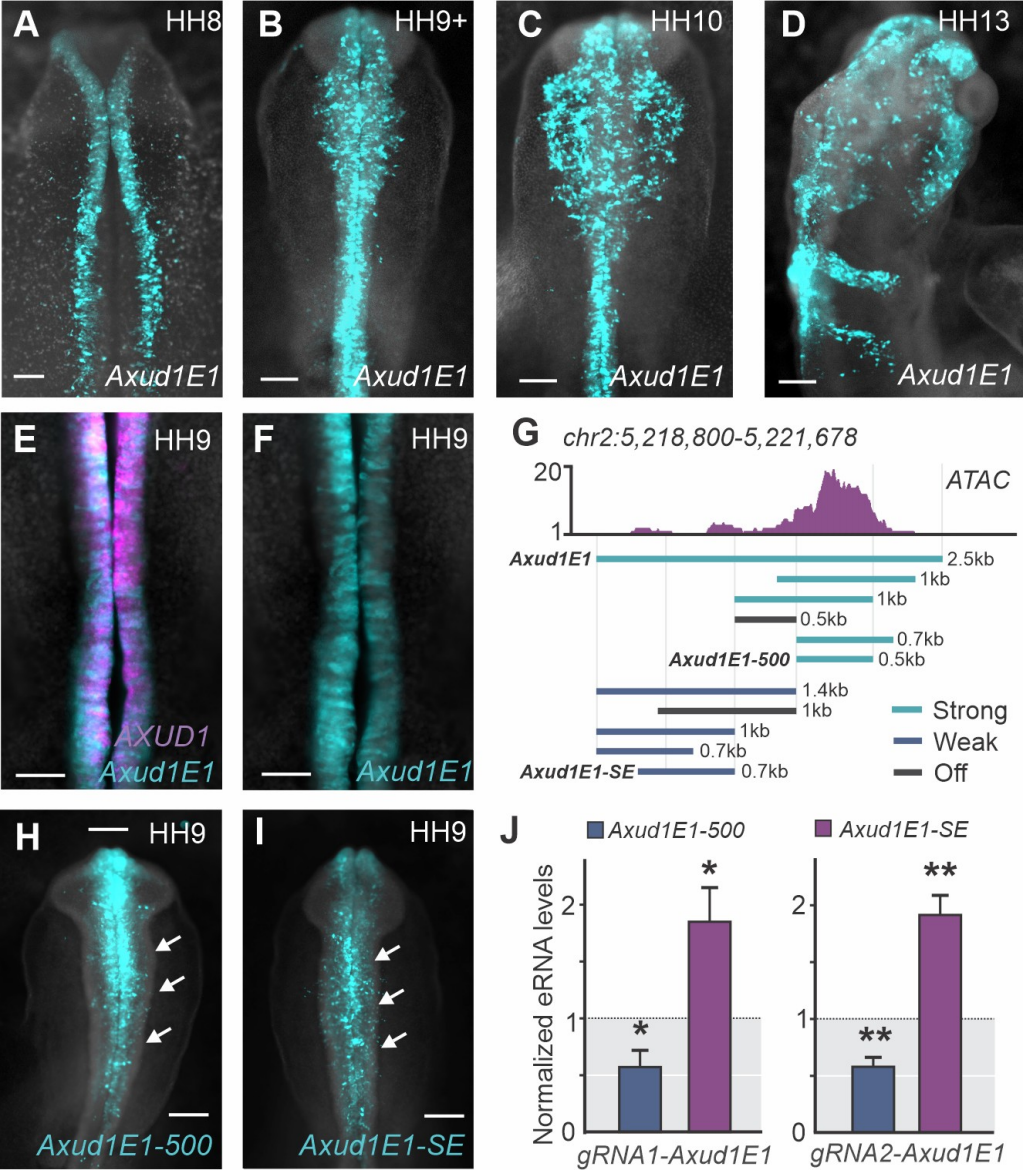
Dissection of the *Axud1E1* enhancer. **(A-D)** Transient transgenesis expression pattern of *Axud1E1* depicting robust activity during neural crest specification (**A**), and in pre-migratory (**B**), early (**C**) and late migratory neural crest cells (**D**). **(E-F**) Double fluorescent *in situ* hybridization for *AXUD1* (magenta) and *eGFP* (*Axud1E1*, turquoise) in transgenic embryos shows colocalization of the endogenous gene (**E**) and the enhancer reporter (**F**). **(G**) ATAC-seq profile at *Axud1E1* and enhancer dissection strategy. Turquoise bars represent enhancer variants that were able to generate strong reporter activity in transgenic embryos. Blue and gray bars represent weak and inactive enhancer variants, respectively. **(H-I)** Dorsal view of embryos transfected with constructs containing *Axud1E1-500* and *Axud1E1-SE* (*Axud1E1* shadow enhancer) defined in (**G**). *Axud1E1-500* displays robust activity in neural crest cells (arrows in **H**). The *Axud1E1-SE* region displays weaker activity in the same cell population (arrows in **I**). **(J)** Quantification of fold change in *Axud1E1-500* and *Axud1E1-SE* eRNA levels when *Axud1E1-500* is targeted with specific gRNAs. Error bars represent ± SEM. Statistical significance determined via an unpaired t-test. HH, Hamburger and Hamilton. Scale bars represent 200μm (**A-D, E-F, H-I**). *p < 0.05, **p < 0.01.

In order to establish the temporal pattern of *Axud1E1* activity, we next examined transgenic embryos at different stages of neural crest development. We detected specific signal from the *Axud1E1* reporter construct in both pre-migratory (Fig 2A) and migratory neural crest (Fig 2B–2D). Double fluorescent *in situ* hybridization (FISH) for *AXUD1* and *eGFP* in transgenic embryos showed that prior to migration, the enhancer drives reporter expression only in neural crest cells that express *AXUD1* (Fig 2E and 2F). Since *AXUD1* is rapidly downregulated after neural crest cells delaminate and begin to migrate [13], we examined transcription of the reporter construct at later developmental stages. FISH for eGFP at HH11 showed that late migratory neural crest cells display no transcription of the reporter gene (S2A Fig). This indicates that the labeling of this cell population with eGFP (Fig 2D) is likely due to the stability of eGFP protein [21]. These results show that *Axud1E1* activity accurately recapitulates the spatial and temporal expression of the endogenous gene during early neural crest development.

To understand the regulation of *Axud1E1*, we performed a series of deletions in the original reporter construct (Fig 2G). We found that the original 2.5kb fragment contained two smaller elements capable of driving reporter activity in neural crest cells (Fig 2H and 2I). The first element (*Axud1E-500*, located in the 3' region of the original construct) spanned 500bp, and was highly active in *AXUD1*+ cells (Figs 2H and S2B). Chromatin immunoprecipitation (ChIP) confirmed that *Axud1E-500* was associated with H3k27Ac and TFAP2A (S2C Fig). The second element (*Axud1E1-SE*), located at the 5' region of *Axud1E1*, displayed similar but weaker activity (Figs 2I and S2D). Since both elements are active in the same cells (S2E and S2F Fig) and *Axud1E1-SE* is located in a region of closed chromatin (Fig 2G), we hypothesized that the latter acts as a shadow enhancer [22]. To test this, we first examined the activity of both cis-regulatory elements in FACS sorted neural crest cells. Quantification of eRNA with qPCR showed specific activity of *Axud1E-500*, while *Axud1E-SE* was transcriptionally silent (S2G Fig). Next, we assessed the existence of a compensatory mechanism between the two enhancers by disrupting *Axud1E-500* with CRISPR/Cas9. We electroporated the right side of chick embryos with a CAS9 expression vector and gRNAs targeted to the *Axud1E-500* [23], whereas the left side was transfected with CAS9 and a control gRNA. eRNA quantification with qPCR showed that targeting of *Axud1E1-500* with two different gRNAs results in a significant activation of *Axud1E-SE* (Fig 2J) in neural crest cells. These results show that *AXUD1* is regulated by a robust tissue-specific enhancer (*Axud1E-500*) that is in close proximity to a shadow regulatory element.

### Wnt effectors LEF1/CTNNB1 directly regulate AXUD1

**Fig 3:**
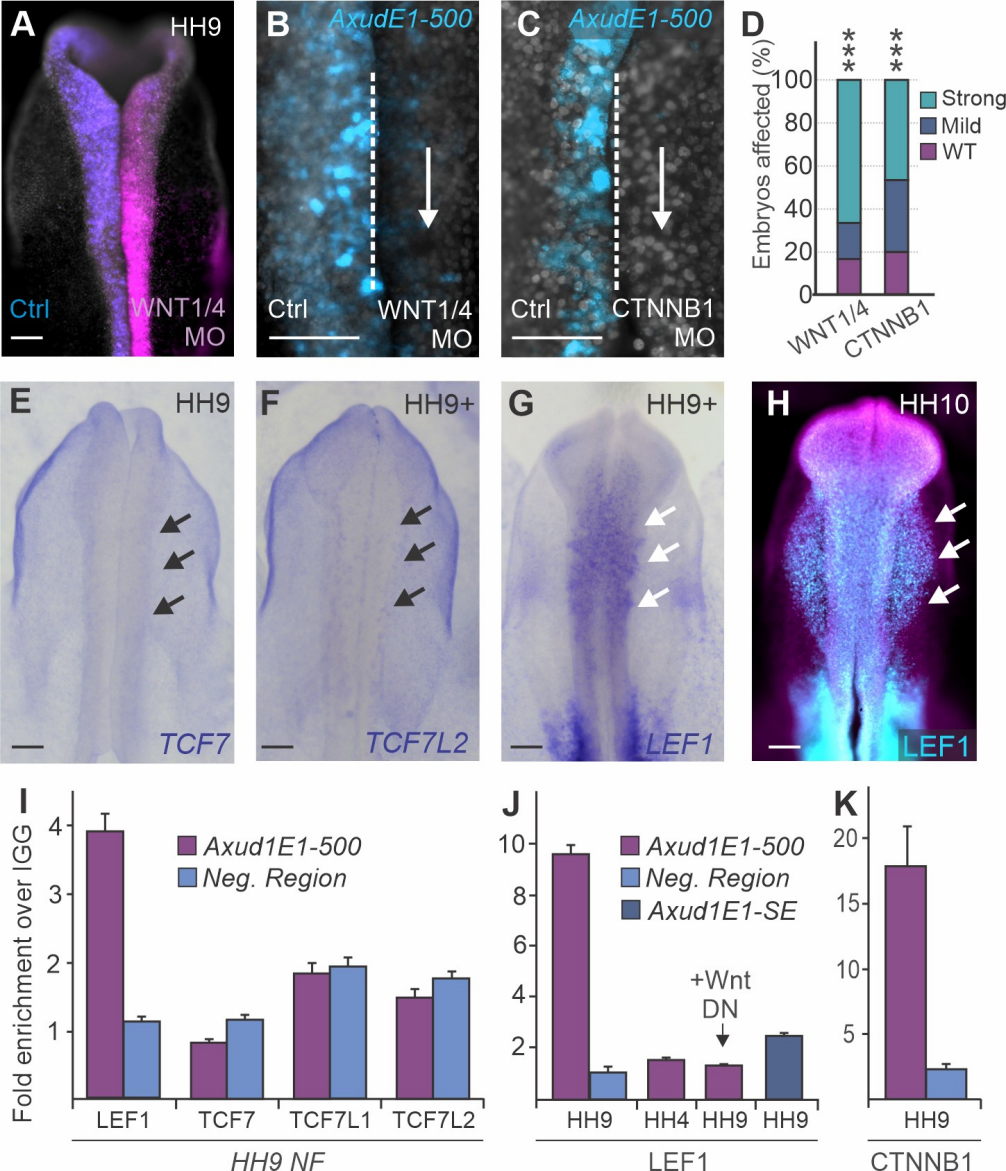
*Axud1E1* is directly regulated by nuclear effectors of canonical Wnt signaling. **(A-C)** *Axud1E1* responds to Wnt pathway manipulation. Dorsal view of an embryo electroporated with control (left) and WNT1/4 (right) morpholinos (**A**) and representative images showing the loss of *Axud1E1-500* activity upon WNT1/4 (**B**) or CTNNB1 (**C**) knockdown in the right side of the embryos. **(D)** Quantification of the effect of WNT1/4 (n = 12) and CTNNB1 (n = 15) loss-of-function assays on *Axud1E1*. Statistical significance determined via ANOVA. **(E-G)** Whole embryo *in situ* hybridization for *TCF7* (**E**), *TCF7L2* (**F**) and *LEF1* (**G**) shows that *LEF1* is the only Wnt nuclear effector robustly expressed in cranial regions during neural crest specification and early migration stages (arrows). **(H)** Immunohistochemistry for LEF1 depicting enrichment of the protein in migrating neural crest cells (arrows). **(I-K)** Wnt effectors CTNNB1 and LEF1 directly bind to *Axud1E1*. Chromatin immunoprecipitation for LEF1, TCF7, TCF7L1 and TCF7L2, performed with neural folds of WT embryos, shows that LEF1 is the only Wnt nuclear effector that co-immunoprecipitates *Axud1E1*. Interaction between LEF1 and *Axud1E1* (**I**) was not detected in neural plate border tissue dissected from gastrula stage embryos (HH4), and was lost upon treatment with a Wnt1 dominant negative (DN) construct (**J**). *Axud1E1-SE* did not interact with LEF1 (**J**). ChIP with a CTNNB1 antibody also revealed robust interaction with *Axud1E1* (**K**). Error bars represent ± SEM. HH, Hamburger and Hamilton; MO, morpholino; NF, neural fold; DN, dominant negative. Scale bars represent 200μm (**A-C, E-H**). ***p < 0.001.

Our previous studies identified *AXUD1* as a downstream target of canonical Wnt signaling, acting as a secondary effector of the pathway during neural crest formation [13]. Thus, we tested whether Wnts directly regulate this transcription factor via *Axud1E1-500*. To define if the enhancer responds to Wnt signaling, we disrupted the pathway in transgenic avian embryos transfected with the *Axud1E1-500* reporter construct. For these experiments, we transfected embryos with a control morpholino on the left side, and morpholinos targeting components of the Wnt signaling pathway on the right side (Fig 3A). We found that knockdown of either the WNT1/4 ligands (Fig 3B) or CTNNB1 (Fig 3C) resulted in a robust loss of enhancer activity (Fig 3D), suggesting Wnt signaling directly regulates *Axud1E1*. To further characterize this interaction, we next identified which Wnt nuclear effector cooperates with CTNNB1 to mediate the pathway’s activity in neural crest cells. We examined the expression of the three TCF/LEF paralogs that promote gene expression downstream of Wnt signaling (*TCF7*, *TCF7L2* and *LEF1*). *In situ* hybridization showed that *LEF1* is strongly enriched in the neural crest during specification, whereas we were unable to detect discernable expression patterns for *TCF7* and *TCF7L2* in the head of avian embryos (Fig 3E and 3G). Immunohistochemistry for LEF1 was consistent with the *in situ* hybridization analysis, showing robust and specific expression of the protein in the neural crest (Fig 3H, S3 Fig).

These results suggest that LEF1 is the main effector of Wnt signaling in neural crest cells. They also indicate that the tissue-specific regulation of Wnt nuclear effectors may play an important role in the activation of target genes. To examine the regulation of *Axud1E1-500* by Wnt nuclear effectors, we conducted a series of ChIP experiments in dissected HH9 dorsal neural folds. While pulldown of LEF1 resulted in co-immunoprecipitation of *Axud1E1-500*, the other TCF/LEF paralogs (TCF7, TCF7L1 or TCF7L2) failed to interact with the enhancer (Fig 3I). Next, we examined whether the interaction between LEF1 and *Axud1E1-500* was stage-specific and dependent on the activation of Wnt signaling. We found that ChIP performed on lateral ectodermal tissue at gastrula stages (HH4) or on neural folds (HH9) transfected with a Wnt dominant-negative construct [16] showed no interaction between LEF1 and the enhancer (Fig 3J). Furthermore, the shadow enhancer *Axud1E1-SE*, which is not accessible (Fig 2G) or active (S2G Fig), was not associated with LEF1 (Fig 3J). Finally, we also found a robust association between CTNNB1 and *Axud1E1-500* (Fig 3K). These results indicate that LEF1 regulates *AXUD1* by interacting with a tissue-specific enhancer and is the main effector of canonical Wnt signaling in neural crest cells.

### *Axud1E1* integrates multiple upstream inputs to regulate gene expression

**Fig 4:**
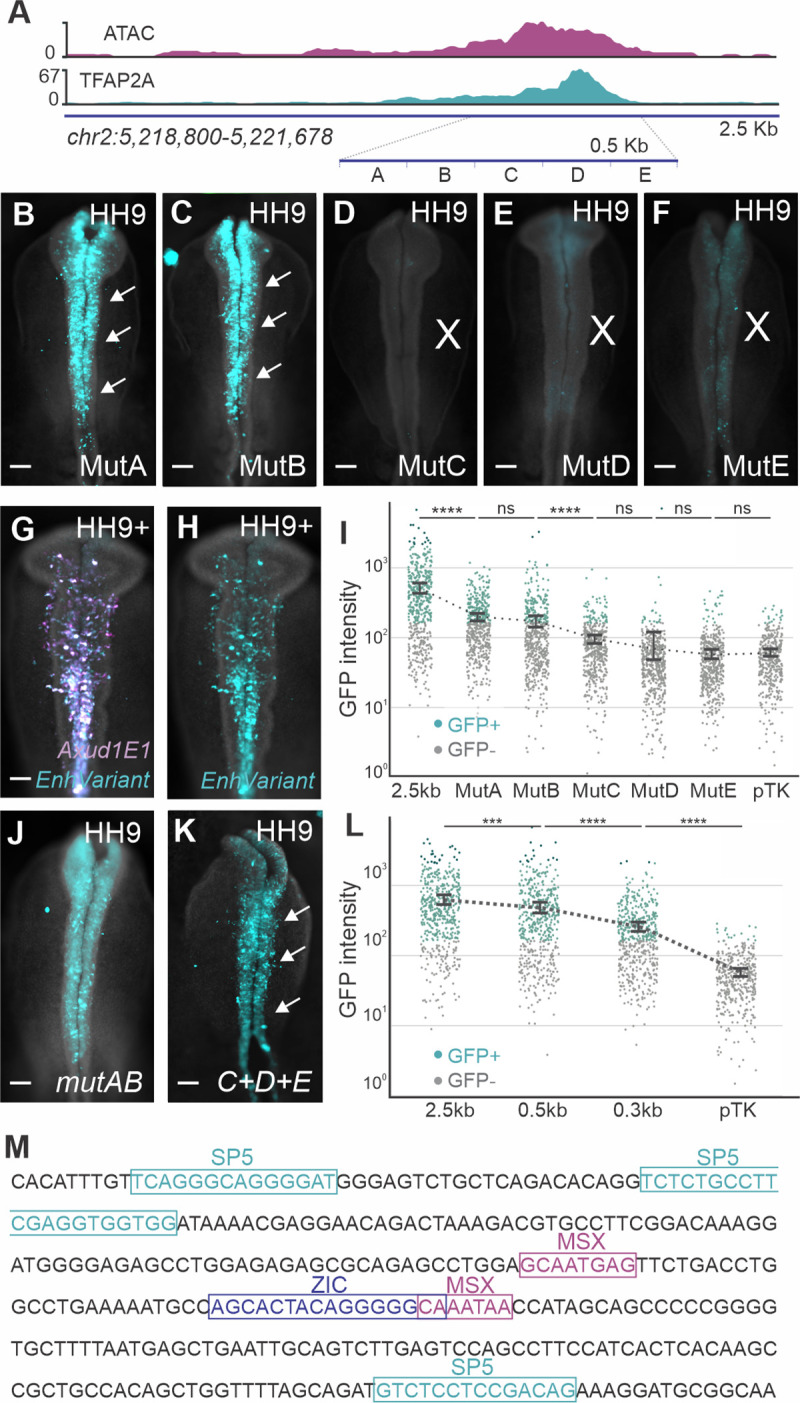
Mutation analysis of *Axud1E1* reveals putative upstream regulators. **(A)** ATAC-seq and TFAP2A occupancy profiles at *Axud1E1*. The blue horizontal bar describes the 100bp regions of *Axud1E1-500* mutated in this analysis. **(B-F)** Transient transgenesis of *Axud1E1-500* mutants shown in (**A**). Mutations in regions A (**B**) and B (**C**) do not eliminate enhancer activity (arrows). In contrast, enhancer activity in neural crest cells is drastically reduced in mutants C (**D**), D (**E**) and E (**F**). **(G-H)** Co-expression strategy for enhancer-reporter quantification. Transient transgenic embryo co-electroporated with the control *Axud1E1* cloned into the vector pTK-mCherry (**G**) and the enhancer variant cloned into the pTK-eGFP vector (**H**). Cranial region of transgenic embryos were dissected and 500 Cherry+ cells were analyzed with flow cytometry. **(I)** Categorical scatterplot depicting the reporter intensity of *Axud1E1-500* mutant constructs as measured by flow cytometry. Statistical significance determined via an unpaired two-tailed t-test. **(J)** Transient transgenesis shows activity of an *Axud1E1-500* variant in which both regions A and B were mutated. **(K)** Transient transgenesis of *Axud1E1-300* shows robust reporter expression in neural crest cells (arrows). **(L)** Categorical scatterplot depicting reporter intensity in *Axud1E*1, *Axud1E*1-500 and *Axud1E*1-300. The statistical significance was determined via an unpaired two-tailed t-test. **(M)** Sequence of *Axud1E1-300* highlighting the MSX1, ZIC1 and SP5 binding motifs defined using JASPAR database. Kb, kilobase; HH, Hamburger and Hamilton; Scale bars represent 100μm (**B-H**, **J-K**); ***p < 0.001; ****p < 0.0001; ns, not significant.

Enhancer analysis is a powerful tool for the identification of novel GRN interactions. To elaborate on the genetic circuit that controls *AXUD1* expression, we further dissected the *Axud1E1-500* enhancer. We divided this element into five 100bp segments (A-E), which were mutated by replacing the endogenous sequence with a 100bp segment of the coding sequence of eGFP (Fig 4A) [24]. We found that mutations in segments A and B did not disrupt enhancer activity in neural crest cells (Fig 4B and 4C). In contrast, mutations in segments C, D and E resulted in a strong loss of reporter expression (Fig 4D and 4F). We next quantified the effects of each mutation using flow cytometry. Embryos were co-electroporated with the original 2.5kb *Axud1E1* enhancer driving expression of mCherry, and each of the mutant constructs upstream of eGFP (Fig 4G and 4H). We then dissociated transgenic embryos and quantified eGFP expression in mCherry-positive cells. We observed that the A and B mutants were still active in neural crest cells, despite a reduction in eGFP expression. Activity of the C, D and E mutants was indistinguishable from the negative control vector (Fig 4I).

**Fig 5:**
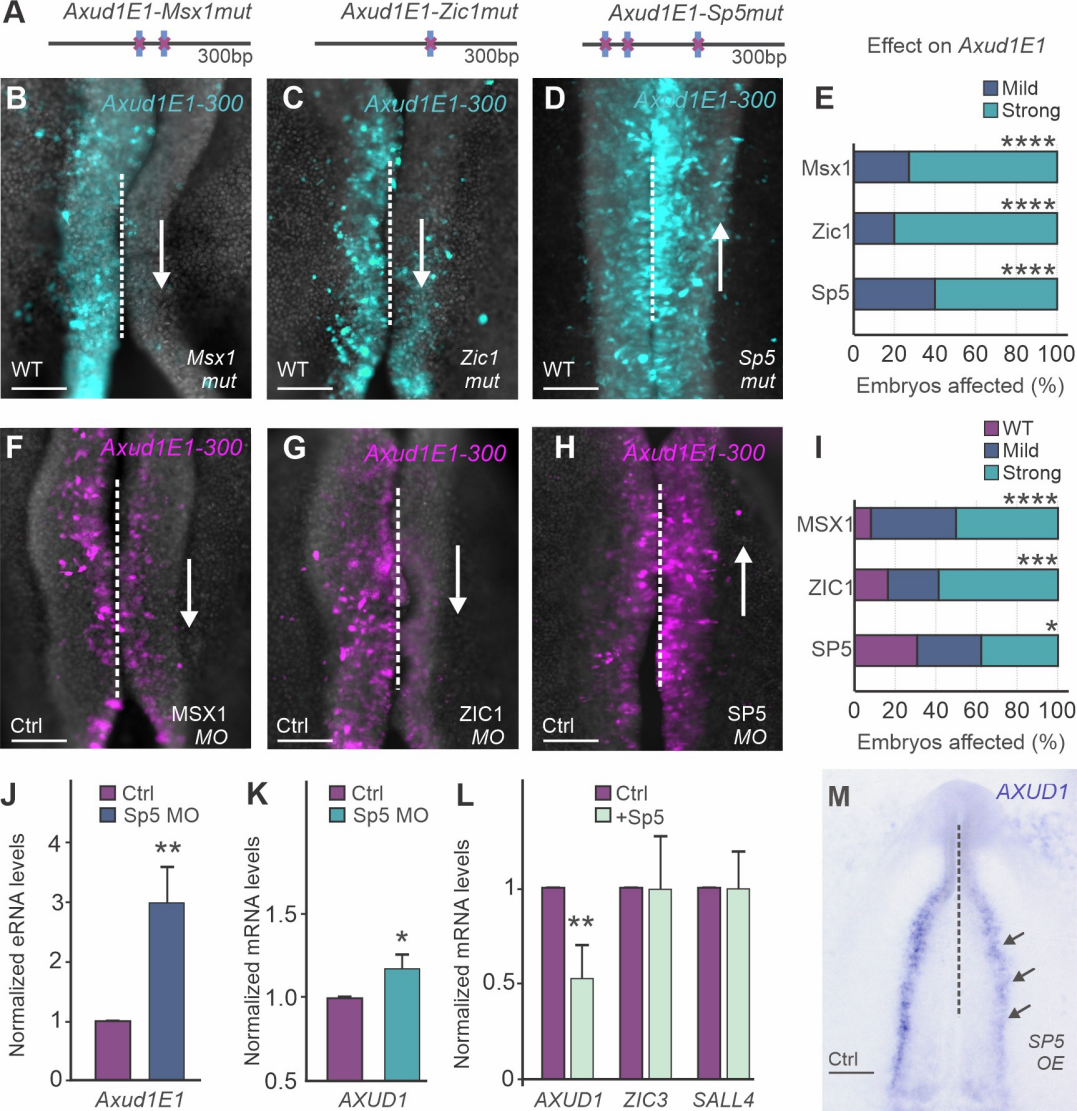
*Axud1E1* is regulated by neural plate border genes and multipotency factor SP5. **(A)** Schematic representation of *Axud1E1-300* mutants. Blue vertical bars represent MSX1, ZIC1 or SP5 binding motifs (see Fig 4M) that were disrupted in each construct. **(B-D)** Electroporation of the *Axud1E1-300* mutants. Embryos were transfected with the wild-type enhancer on the left side and a mutant construct on the right side. Reduction of enhancer activity was observed when MSX1 (arrow in **B**) and ZIC1 (arrow in **C**) binding motifs were mutated. In contrast, mutation of SP5 motifs (**D**) resulted in an increase of *Axud1E1-300* activity (upward arrow). **(E)** Quantification of the effect of MSX1 (n = 11), ZIC1 (n = 10) and SP5 (N = 10) binding site mutation on *Axud1E1-300* activity. Statistical significance determined via ANOVA. **(F-H)** Effect of knockdown of putative regulators MSX1 (**F**), ZIC1 (**G**) and SP5 (**H**) on the activity of *Axud1E1-300*. MSX1 and ZIC1 knockdown result in reduction of *Axud1E1-300* activity. SP5 morphant embryos display an increase in reporter expression indicating that SP5 represses *Axud1E1*. **(I)** Quantification of the effect of MSX1 (n = 12), ZIC1 (n = 12) and SP5 (N = 16) loss-of-function on *Axud1E1-300*. Statistical significance determined via ANOVA. **(J-K)** SP5 loss-of-function results in premature activation of *Axud1E1* and *AXUD1*. qPCR for *Axud1E1* (**J**) and *AXUD1* (**K**) transcripts (HH6) in control neural plate border tissue vs neural plate border tissue transfected with SP5 morpholino. Error bars represent ± SEM. The statistical significance was determined via an unpaired t-test. **(L)** qPCR for *AXUD1*, *ZIC3* and *SALL4* transcripts in control vs neural folds transfected with an SP5 overexpression construct. Error bars represent ± SEM. The statistical significance was determined via an unpaired t-test. **(M)** *In situ* hybridization for *AXUD1* after overexpression of SP5 within the right side of the embryo depicts reduction in *AXUD1* expression levels (arrows). HH, Hamburger and Hamilton; mut, mutant; MO, morpholino; WT, wild type; bp, base pair; Scale bars represent 100μm (**B-D**, **F-H**), 500μm (**M**); *p < 0.05; **p < 0.01; ***p < 0.001; ****p < 0.0001.

This analysis suggests that segments C,D, and E of *Axud1E1-500* are critical for enhancer activity. Consistent with this, a reporter construct containing the simultaneous mutations in A and B is active in neural crest cells (Figs 4J and S4A). We thus generated a 300bp version of the enhancer containing only segments C, D and E. This construct (*Axud1E1-300*) (Fig 4K and S4B Fig), was able to drive specific eGFP expression (Fig 4L). Next, we screened this 300bp enhancer core using the JASPAR database to identify binding sites of potential upstream regulators. We found motifs for genes expressed in the neural crest lineage, like MSX1 and ZIC1, and for the Wnt target gene and multipotency factor SP5 (Fig 4M). To assess the importance of these motifs, we generated versions of the enhancer carrying mutations in these binding sites (Fig 5A and S2 Table) for transgenic reporter assays. Enhancer variants were transfected on the right side of chicken embryos, whereas the wild-type enhancer was transfected on the left side. While mutation of the MSX1 and ZIC1 motifs resulted in loss of enhancer activity (Fig 5B, 5C and 5E), loss of SP5 binding sites drastically increased enhancer output (Fig 5D and 5E). These findings suggest that MSX1 and ZIC1 are activators of *Axud1E1* while SP5 acts as a repressor of the enhancer.

To confirm the roles of neural plate border genes (*MSX1* and *ZIC1*) and the multipotency factor SP5 in the regulation of *AXUD1*, we performed loss-of-function experiments for these transcription factors. Gastrula-stage embryos (HH4) were electroporated with control and targeted morpholinos and incubated until HH9. MSX1 and ZIC1 morphants exhibited a reduction in reporter expression (Fig 5F and 5G). In contrast, knockdown of SP5 resulted in an increase in enhancer activity (Fig 5H and 5I). Furthermore, the SP5 morphant side of the embryos displayed a robust upregulation of the endogenous *Axud1E1* (Fig 5J) and premature transcription of *AXUD1* at HH6 (Fig 5K). To confirm that SP5 acts as a repressor of *AXUD1*, we overexpressed SP5 using pCI-H2B-RFP [25]. Analysis of dorsal neural folds from single embryos (left vs. right) with RT-PCR and *in situ* hybridization showed a significant reduction in the expression of *AXUD1* (Fig 5L and 5M) following SP5 overexpression.

Finally, we examined the regulation of *Axud1E1* accessibility. *Axud1E1* is associated with TFAP2A (Figs 1E and S2C), a pioneer transcription factor involved in chromatin remodeling during neural crest specification [17]. Despite this, we found that mutation of several TFAP2A binding sites in the *Axud1E1* did not affect the activity of the enhancer in reporter assays (S5C Fig). This led us to postulate that TFAP2A regulates *Axud1E1* at the chromatin level. Consistent with this, we found that knockdown of the pioneer factor caused a significant reduction in accessibility of the endogenous enhancer (S5D Fig), as assayed by ATAC-qPCR. Taken together, these data indicate that *AXUD1* is regulated by a combination of activating and inhibitory inputs, where TFAP2A promotes chromatin accessibility, while MSX1 and ZIC1 act as bona fide transcriptional inputs. In contrast, SP5, which is also directly regulated by canonical Wnt signaling [26], represses *AXUD1* via *Axud1E1*.

### SP5 directly regulates components of the neural crest GRN

**Fig 6:**
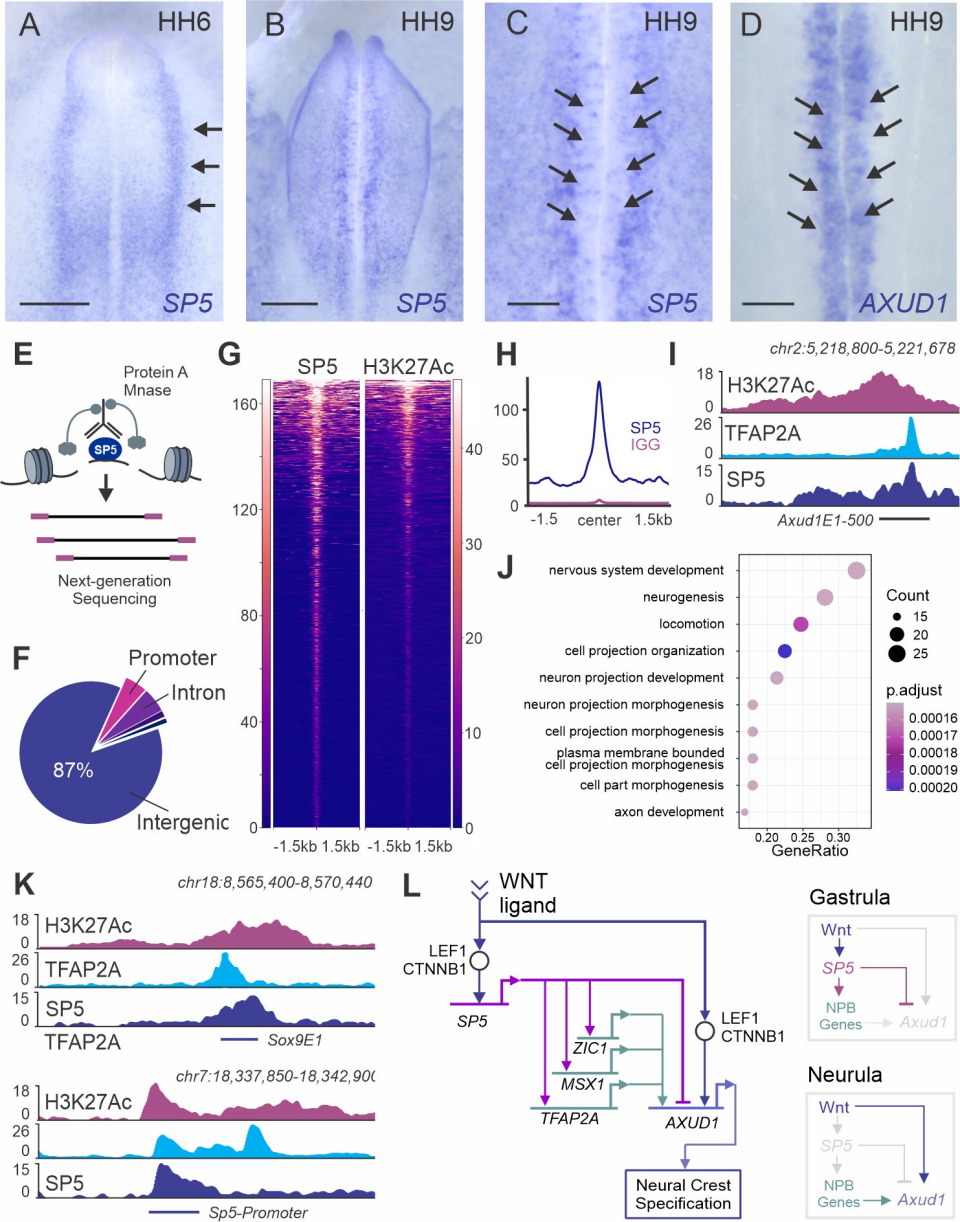
SP5 interacts with enhancers of multiple neural crest genes. **(A-B)** *SP5* is transiently expressed in the neural crest lineage. Whole mount *in situ* hybridization shows expression of *SP5* in the neural plate border (arrows in **A**). During neural crest specification, *SP5* is excluded from the midline of the embryo (**B**). **(C-D)** Whole mount *in situ* hybridization shows that *SP5* and *AXUD1* are expressed in complementary expression patterns during neurula stages (HH9). *AXUD1* transcripts are detected in the pre-migratory neural crest cells (**D**). **(E)** Diagram of CUT&RUN experiment used to map genome occupancy of SP5. **(F)** Genomic locations of SP5 binding events. The majority of SP5-associated regions are intergenic. **(G)** Heatmaps displaying SP5 and H3K27Ac signal at SP5-bound regions. **(H)** CUT&RUN profiles showing binding of SP5 and normal rabbit IgG at SP5-bound peaks. **(I)** CUT&RUN profiles of H3K27Ac, TFAP2A and SP5 at the *Axud1E1-500* region. **(J)** Significantly enriched GO terms from Gene Ontology analysis of genes associated with SP5-bound regions. The diagram includes GO terms with Gene Ratio > 0.1. **(K)** CUT&RUN profiles of H3K27Ac, TFAP2A and SP5 at the enhancer *Sox9E1* and the *SP5* promoter. **(L)** Sub-circuit controlling the specification program in gastrula- and neurula-stage chicken embryos. *SP5* and *AXUD1* are activated by Wnt signaling. Sequential activation of NPB genes and the direct repression of *Axud1E1* by SP5 restrict the timing of *AXUD1* expression to specification stages. NPB, neural plate border; Kb, kilobase; HH, Hamburger and Hamilton; Scale bars represent 500μm (**A-B**), 200μm (**C-D**).

Our dissection of *Axud1E1* allowed for the delineation of a regulatory circuit that controls *AXUD1* expression, in which Wnt signaling and neural plate border specifier genes act as positive inputs, and SP5 acts as a repressor. To determine how SP5 regulates *AXUD1* during neural crest specification, we examined the expression patterns of these genes using *in situ* hybridization. We found that *SP5* was robustly expressed in the neural plate border at HH6 (Figs 6A and S6A), just prior to the onset of *AXUD1* expression. At specification stages, *SP5* expression is rapidly downregulated in the neural crest lineage (Figs 6B and 6C and S6B–S6D), just as *AXUD1* transcription begins. At this developmental stage, *SP5* transcripts are detected in the non-neural ectoderm (S6C’ Fig), whereas *AXUD1* is restricted to the neural crest (Fig 6D). Consistent with this, double FISH shows co-localization of *SP5* and neural plate border *PAX7* (S6E Fig), whereas we found no overlap between the former and neural crest marker *TFAP2B* at migration stages (S6F and S6G Fig). The temporal segregation in the expression of *AXUD1* and *SP5* is also evident in their regulation by canonical Wnt signaling. ChIP for CTNNB1 shows that the binding of the nuclear effector to *Axud1E1* significantly increases from HH5 to HH9, whereas association to a *SP5* enhancer follows the opposite trend (S7A Fig).

To confirm that SP5 directly regulates *Axud1E1*, and to gain further insight into its function, we mapped the occupancy of this transcription factor using Cleavage Under Targets and Release Using Nuclease (CUT&RUN) [27]. This technique relies on a protein A-Mnase fusion protein, which binds to an antibody of choice and cleaves DNA fragments associated with the targeted transcription factor (Fig 6E). CUT&RUN analysis in neural folds dissected from HH7 embryos revealed 1016 SP5-bound regions, the majority of which were located in intergenic regions (87%) (Fig 6F). Motif enrichment analysis of these peaks identified a GC-rich motif common to Sp/Klf zinc-finger transcription factors [28] (pValue = 1e-155) (S7B Fig). SP5 peaks were above background levels, and positive for H3K27Ac (Fig 6G and 6H), indicating that SP5 interacts with active enhancers. Consistent with our enhancer dissection (Fig 5), we also observed robust association of the transcription factor with *Axud1E1* (Fig 6I).

The results from our genomic analysis indicate SP5 regulates a large number of genes in the neural crest lineage. Gene ontology analysis shows SP5 targets are involved in many developmental processes like nervous system development, neurogenesis, and morphogenesis (Fig 6J). Our results are also consistent with reports that SP5 regulates its own transcription, as we detect strong occupancy in the *SP5* gene promoter (Fig 6K). Other neural crest genes, including *SOX9* (Fig 6K), *TFAP2A*, *ZIC1*, and *MSX1* also contained SP5 peaks in active enhancers in their *loci* (S7D Fig). Taken together, our results allow for the assembly of a regulatory sub-circuit that controls sequential activation of Wnt target genes during neural crest development (Fig 6L).

## Discussion

Cis-regulatory analysis has served as a powerful tool to decode the genetic programs that control metazoan development [29]. Dissection of tissue-specific elements can uncover how multiple inputs are processed to generate specific domains of gene activity. Previous studies on neural crest enhancers have resulted in the identification of new genes involved in the formation of this cell type [24,25,30–32]. Tissue-specific enhancers have also been useful for labeling and isolating pure populations of neural crest cells for genomic analysis [7,33,34]. Whereas previous studies have employed evolutionary conservation, histone marks or accessibility, our results indicate that chromosome conformation capture is a viable strategy for rapid identification of cis-regulatory elements. The subsequent dissection of *Axud1E1* revealed an intricate combination of upstream inputs, which included effectors of signaling systems, multiple neural plate border genes and both positive and inhibitory inputs. The complexity of *AXUD1* regulation was further underscored by the existence of a shadow enhancer adjacent to the core of *Axud1E1*. These results support our previous suggestion that *AXUD1* occupies an important position in the neural crest GRN and is a direct target of Wnt signaling [13].

Comparative analyses of developmental GRNs show that these genetic programs are composed of collections of sub-circuits, which are responsible for executing well-defined tasks [11]. Here we outline a sub-circuit centered on Wnt signaling and effector genes *SP5* and *AXUD1*, which supports the sequential activation of regulatory modules in the neural crest GRN. The sub-circuit (Fig 6L) is structured as an incoherent feedforward loop [35], in which Wnts activate both *SP5* [26,36] and *AXUD1*, while SP5 represses *AXUD1* by interacting with a tissue-specific enhancer. These types of motifs are termed incoherent since the upstream signal (Wnts) directly activates a target gene (*AXUD1*) but also promotes the expression of a repressor (*SP5*) of said gene. We propose that the sub-circuit relies on repressive interactions between Wnt target genes to promote temporally distinct phases of gene expression. The inhibitory interaction between SP5 and *AXUD1* prevents these factors and their downstream targets from being expressed at the same time. Since SP5 activates neural plate border genes [37] and AXUD1 promotes the expression of specification genes [13], this regulatory architecture prevents any overlap between these two GRN modules.

Temporal operation of the sub-circuit involves the transition between the two states shown in Fig 6L. The activation of *AXUD1* and the specification program depends upon a reduction in the levels of SP5 and on the presence of TFAP2A, MSX1 and ZIC1. Curiously, neural plate border genes are also regulated by SP5 [37], which directly interacts with tissue-specific enhancers located at their *loci* (S6D Fig). This dual role of SP5, as both an activator (of neural plate border genes) and a repressor (of specification genes) is consistent with studies that have shown that it is able to exert both functions [36,38]. We postulate that once SP5 promotes the neural plate border program, the expression of these genes is stabilized by numerous positive feedback loops [4]. SP5 is subsequently downregulated, which releases repression of *AXUD1* and allows specification to occur. Like other multipotency factors, SP5 expression decreases during avian neural crest development (S7D Fig), presumably due to a reduction in levels of Wnt activity [39] (in *Xenopus*, expression of SP5 can be detected in migratory cells, but there is still a reduction in expression of the gene [37]). Nonetheless, two features of this sub-circuit control the separation of the neural plate border and specification programs. First, SP5 needs to activate *TFAP2A*, *MSX1* and *ZIC1* before these genes can promote *AXUD1* expression. Second, the direct repression of *AXUD1* by SP5 prevents the former from being transcribed prematurely.

These results highlight the importance of repressive interactions in the neural crest GRN. A significant shortcoming of the current version of the network is that it is built primarily of positive regulatory links; only a small number of studies have focused on transcriptional repressors, like *SNAI2* [40]. This is likely a consequence of technical limitations (identifying inhibitory interactions is technically challenging) but also because the importance of repression in GRNs has often been underestimated. Genomic analysis has demonstrated that cell fate specification programs rely heavily on inhibitory interactions, which act to silence alternative fates [41], or to promote stepwise activation of gene expression. Additional cis-regulatory analyses or combinations of genomic approaches [42] will be required to delineate the role of repression in the neural crest GRN. Our findings suggest the existence of a set of inhibitory interactions that control the timing of developmental transitions during neural crest development. Elaboration of network circuitry and identification of additional sub-circuits will be necessary to shed further light on the molecular logic of neural crest development.

## Methods

### Ethics statement

All experiments were performed in accordance with Memorandum of Understanding and Agreement 16274–1, which was approved by the Institutional Biosafety Committee of Cornell University.

### Collection of chick embryos

Fertilized Leghorn White chicken eggs were obtained from the Department of Animal Science, University of Connecticut. Eggs were incubated at 37°C until embryos reached the desired developmental stages. Embryos were collected and cultured according to the EC protocol [43], and staged using the Hamburger and Hamilton staging system [44].

### *In Situ* Hybridization

For *in situ* hybridization, embryos were fixed in phosphate buffer saline (PBS) containing 4% paraformaldehyde (PFA) for 2 hours at RT. Following fixation, embryos were dissected, washed with PBST, dehydrated and stored in methanol at −20°C. Whole-mount *in situ* hybridization was performed as previously described [45]. For double *in situ* hybridization, we used the Tyramide TSA system from Perking Elmer (TSA Plus Cyanine 5 and Fluorescein, NEL754001KT) as previously described [46].

### 3C-qPCR

Chromosome Conformation Capture (3C) libraries were generated based on previously published protocols [47–49] with modifications to account for lower amounts of starting material. Neural folds were dissected from HH9 embryos (n = 100–120 per replicate) in Ringers solution and dissociated in Accumax (Innovative Cell Technologies, #AM105). After crosslinking, cells were washed in cold PBS complemented with protease inhibitors, resuspended in Lysis Buffer and kept on ice for 10 min. Lysed cells were then incubated in 1.2x CutSmart (New England Biolabs) restriction buffer and incubated for 1h at 37°C with 0.3% SDS. After addition of 2% Triton X-100 lysed cells were re-incubated for 1h at 37°C. DNA digestion was performed with 400UI NCOI (New England Biolabs, #R3193) at 37°C overnight. After enzyme inactivation with 1.6% SDS and overnight incubation at 65°C, cells were transferred to 1.15x ligation buffer (New England Biolabs, #M0202) and incubated with 15% Triton X-100 for 1h at 37°C. Ligation was performed for 4h at 4°C with T4 DNA ligase (New England Biolabs, #M0202). Eluted DNA was treated with 10mg/ml Proteinase K overnight at 65°C. After RNase treatment, samples were purified with phenol-chloroform, concentrated using Amicon Ultra-0.5 ml 30k columns (Millipore, #UFC5030BK) and quantified in Qubit. A control library was assembled by mixing equimolar amounts of PCR products spanning the *AXUD1* locus with minimal overlap. Amplification efficiency (slope and intercept) of primers pairs (fragment primers plus constant primer) was verified via qPCR of 10x dilutions of digested/ligated control library [50]. Interaction frequencies were determined by the normalization of ligation products/loading control (*GAPDH*) as described in [50] (S3 and S4 Tables). The 3CqPCR primers are described in S5 Table.

### Quantitative reverse transcription PCR (RT-PCR)

eRNA expression levels were determined through RT-PCR in microdissected neural crest cells. The tissue was lysed in lysis buffer from RNAqueous-Micro Total RNA Isolation Kit (ThermoFisher, #AM1931) and the total RNA was isolated following manufacturer’s protocol. cDNA was then synthesized using SuperScript III Reverse Transcriptase and Random Primers (ThermoFisher, #18080051) according to the kit’s protocol. RT-PCR was performed using Power Sybr Green PCR master mix (Thermo Fisher, 4368577) in an ABI viia7 RT-PCR machine. eRNA levels at the *AXUD1* locus are presented normalized to reference gene *HPRT1*.

To quantify changes in gene expression caused by SP5 loss-of-function or gain-of-function, we microdissected single neural folds from control and targeted sides of the embryo, which were subsequently lysed in lysis buffer from Power SYBR Green Cells-to-CT Kit. RNA extraction and cDNA preparation were performed according to the kit's protocol). RT-PCR was performed using Power Sybr Green PCR master mix (Thermo Fisher, 4368577) in an ABI viia7 RT-PCR machine. Ct values of all genes were normalized to reference gene *HPRT1* and expressed as a fold change compared to the control sample. The qPCR primer sequences are listed in S5 Table.

### Transient transgenesis

Enhancer plasmids, morpholinos and expression vectors were transfected in chick embryos at HH4 by *ex ovo* electroporation, as previously described [13]. Constructs were injected between the epiblast and vitelline membrane of embryos at a concentration of 1-2ug/ul and electroporated with platinum electrodes (five 50ms pulses of 5.1V, with an interval of 100ms between pulses). In all gene knockdown and overexpression experiments, the embryos were injected bilaterally with the control reagent on the left side and the targeted reagent on the right side. Following electroporation, embryos were cultured in albumin at 37°C until they reached appropriate developmental stages. Embryo survival was >90% and all embryos were screened to ensure uniform electroporation and proper embryo morphology prior to further downstream analysis.

### Enhancer-reporter assays

Putative enhancers (S1 Table) defined by DNA accessibility and TFAP2A binding profiles in neural crest cells were amplified from HH10 chicken genomic DNA and cloned in the pTK-eGFP vector [19]. Single cell measurements with flow cytometry were performed to quantify the intensity of enhancer variants. Heads from three HH9 embryos co-electroporated with the pTK-eGFP construct and *Axud1E1-2500bp*:*mCherry*, were dissected and dissociated in Accumax (Innovative Cell Technologies, #AM105). 500 cells per sample were analyzed and plotted in categorical scatter plots using Seaborn Python library. ZIC1, MSX1 and SP5 binding sites in *Axud1E1-300* were identified by scanning this sequence using the JASPAR database of transcription factor binding profiles, with a minimal relative profile score threshold of 80% [51]. Mutant constructs (S2 Table) were cloned into pTK-eGFP, and compared to the wild-type enhancer in double-sided electroporations.

### Chromatin immunoprecipitation

For each experiment, chromatin was isolated from 20 cranial neural folds dissected from HH8-9 embryos or 10 anterior neural plate border regions from HH5 embryos. Immunoprecipitation was performed as described [33], using the H3K27Ac (Abcam, #ab177178), TFAP2A (DSHB #3B5), LEF1 (Millipore, #17–604), CTNNB1 (BD Biosciences, #610154), TCF7 (Cell Signaling, #C63D9), TCF7L1 (Cell Signaling, #D15G11) and TCF7L2 (Cell Signaling, #C48H11) antibodies and normal mouse IgGs (Millipore, #17–604) as controls. In WNT dominant negative assays, HH4 embryos were electroporated with a WNT dominant negative construct [16] and at stages HH8-9, cranial neural folds were dissected and processed as described above. The primers used for qPCR quantification are described in S5 Table.

### Immunohistochemistry

For whole-mount immunohistochemistry, embryos were collected at appropriate developmental stages and fixed in 4% PFA-PB for 20 mins at RT. Following fixation, embryos were dissected from the filter paper and washed in TBS containing 0.1% Triton and 1% DMSO (TBTD). Embryos were blocked at RT for 2h in TBTD supplemented with 10% donkey serum and incubated in primary antibody (rabbit anti-LEF1, Abcam, #ab137872; rabbit anti-TFAP2B, Abcam, #ab186424 or mouse anti-NHK1, DSHB, #3H5) diluted in blocking solution, overnight at 4°C. Following the primary antibody incubation, embryos were washed, blocked for 30mins at RT, and stained with appropriate secondary antibodies (Molecular Probes) for 2h at RT. Following the secondary antibody step, the embryos were washed, stained with DAPI and post-fixed with 4% PFA for 1h prior to imaging. Whole-mount images were collected using an upright Zeiss Axio Imager fluorescent microscope.

### Knockdown assays

All loss-of-function assays were performed with double-sided injections of morpholinos and their respective controls to allow single-embryo internal controls. Wnt knockdown was performed by the combined inhibition of WNT 1 and 4 by morpholinos at 1.25uM each, while CTNNB1, SP5, MSX1, ZIC1 and TFAP2A morpholinos were used at 1.5uM (S5 Table). Embryos were incubated until the desired stage and imaged to evaluate enhancer reporter activity.

### SP5 overexpression

For overexpression assays the *SP5* expression constructs were assembled by insertion of the full-length cDNA sequence of avian *SP5* in a pCI-H2B-RFP backbone [52]. The coding sequences were PCR amplified from an HH8 cDNA library. The *SP5* expression construct was electroporated as described above, and paired single neural folds (left side = control, right side = knockdown) were dissected and processed individually for RT-PCR.

### CRISPR-Cas9 mediated enhancer loss-of-function

We employed a CRISPR-Cas9 system optimized for chick embryos to disrupt the activity of *Axud1E1-500* [23]. Two gRNAs were designed using online resources (crispor.tefor.net) and cloned downstream of the U6 promoter in the cU6.3 vector (S5 Table). To assess the effect of endogenous *Axud1E1-500* knockdown, gastrula-stage embryos were electroporated with a pCAGG-nls-Cas9-nls-GFP vector and the *Axud1E1-500* gRNAs. A control gRNA was used on the left side of the embryo. Embryos were re-incubated at 37°C. At stage HH9, embryos were screened for robust GFP expression in both sides, and half heads were dissected for control and targeted sides of the embryo. Pools of three half heads were then dissociated in Accumax (Innovative Cell Technologies, #AM105) for 30 min. After dissociation, cells were resuspended in HANKS solution supplemented with 0.5% BSA. Control and target GFP+ cell suspensions (150–300 cells) were sorted into 50 μl of lysis buffer from the Power SYBR Green Cells-to-CT Kit (ThermoFisher, 4402955) using a BD AriaFusion cell sorter. Samples were processed following the manufacturer’s protocol. *Axud1E1-500* and *Axud1E1-SE* expression levels were determined using RT-PCR as described above. cU6.3 and pCAGG-nls-Cas9-nls-GFP vectors were a gift from Dr. Marianne Bronner.

### ATAC-qPCR

To examine the control of *Axud1E1* accessibility, we performed ATAC-qPCR in embryos transfected with a TFAP2A morpholino. HH4 embryos were bilaterally injected with control and targeted morpholinos, as described previously. Embryos were incubated at 37°C until HH9, when dorsal neural folds were surgically dissected. Paired single neural folds were individually processed for DNA tagmentation following the ATAC protocol described in [17]. For each genomic location, specific enrichment was quantified by RT-PCR (S5 Table). Raw CT values were first normalized to a control region defined by the absence of transcription factor binding, no enrichment of active histone marks and low DNA accessibility in our CUT&RUN and ATAC datasets. Changes in chromatin accessibility in morpholino treated samples were subsequently compared to control samples.

### CUT&RUN

Neural folds were dissected from HH7-8 embryos (n = 20 per CUT&RUN experiment). Cells were dissociated in Accumax for 20min at RT under mild agitation. CUT&RUN experiments were carried out as previously described [17]. Briefly, cells were immobilized on BioMag Plus Concanavalin A magnetic beads (Bangs Laboratories, BP531) and incubated with rabbit anti-Sp5 (Abcam, # ab36593) antibody (1:50) overnight at 4°C. After washing away unbound antibody, protein A-MNase was added to a final concentration of 700ng/mL and incubated for 1h at 4°C. Cells were cooled to 0°C and CaCl2 was added to a final concentration of 2mM to activate the MNase enzyme. MNase digestion was performed for 45min and terminated by the addition of 2XSTOP buffer. The protein-DNA complexes were released by centrifugation and digested with proteinase K for 10 min at 70°C. DNA fragments were isolated via phenol-chloroform extraction and ethanol precipitation. Protein A-MNase was kindly provided by Dr. Steven Henikoff [27].

### CUT&RUN library preparation

CUT&RUN libraries were prepared using the NEBNext Ultra II DNA Library Prep Kit (New England Biolabs, #E7645) following the manufacturer’s protocol. Fragment analysis was performed with ABI 3730xl DNA Analyzer to perform quality control for the libraries. Equimolar concentrations of the libraries were pooled using the KAPA Library Quantification Kit—ROX Low (Roche, #07960336001) and sequenced with paired-end 37bp reads on an Illumina NextSeq500 instrument.

### CUT&RUN data analysis

Paired-end sequencing reads from the CUT&RUN libraries were trimmed using Cutadapt [53]. Reads were filtered for those with a minimum length of 25bp or longer and aligned to the reference chicken Galgal5 assembly using Bowtie2 [54]. Picard MarkDuplicates tool was used to mark duplicate reads and BAM files were filtered with SAMtools to discard unmapped reads (those that were not the primary alignment, reads failing platform/vendor quality checks, and PCR/optical duplicates (-f 2 -F 1804). Peak calling was performed using MACS version 2.1 with a pValue cutoff of 0.01, skipping the shifting model and extending read sizes to 200bp (—nomodel—extsize 200). Representative heatmaps showing the SP5, H3K27Ac and IgG enrichment at SP5 bound peaks were generated using the deepTools2 package [55]. The GO-category analysis was performed with the clusterProfiler R package [56] to assay for over-represented Biological Processes,with a pValue cutoff of 0.05. Motif enrichment analysis was performed using the HOMER findMotifsGenome package [57].

## Supporting information

S1 Table Genomic position of enhancers analyzed in this study. (XLSX)

S2 Table Collection of *Axud1E1* mutations performed during enhancer dissection. (XLSX)

S3 Table Number of biological replicates and pValues for quantitative experiments. (XLSX)

S4 Table Source Data for quantitative experiments. (XLSX)

S5 Table Nucleotide sequences for oligonucleotides or primers used. (XLSX)

S1 Fig Activity of *Axud1E1* in pre-migratory and migratory neural crest cells. **(A)** Immunohistochemistry for the endogenous TFAP2B protein (magenta) in transverse sections of an HH9 transgenic embryo transfected with *Axud1E1*:*eGFP* (turquoise) (**A**). Reporter expression indicates enhancer activity (**A’**) in TFAP2B+ cells located at the dorsal neural folds (**A”). (B)** Immunohistochemistry (transverse sections) for HNK1 marker (magenta) in an HH10 transgenic embryo transfected with *Axud1E1*:*eGFP* (turquoise) (**B**). Arrows indicate specific activity of *Axud1E1* (**B’**) in migratory HNK1+ cells (**B”**). HH, Hamburger and Hamilton; Scale bars represent 50μm.
(TIF)

S2 Fig Supplemental data on the activity of *Axud1E1-500* and *Axud1-SE* in avian embryos. **(A-A’)** Extended *Axud1E1* reporter activity in late migratory neural crest cells is due to GFP stability. (**A**) Transient transgenesis expression pattern of Tfap2aE1:mChe (magenta) and Axud1E1:GFP depicting *Axud1E1* reporter activity in late migratory neural crest cells (arrows). (**A’**) Same embryo presented in (**A**) after double fluorescent *in situ* hybridization targeting *mcherry* and *gfp* indicated reduced expression of *Axud1E1* in late migratory cells (arrows). **(B)** Double fluorescent *in situ* hybridization for *AXUD1* and *eGFP* in transgenic embryos shows colocalization (arrows) of the endogenous gene (magenta) and the enhancer reporter (GFP, *Axud1E1-500*, turquoise) (**B’**). **(C)** Representative ChIP-qPCR experiment for the active chromatin mark H3K27ac and the neural crest pioneer factor TFAP2A indicates that *Axud1E1* is an active neural crest enhancer. **(D)** Double fluorescent *in situ* hybridization for *AXUD1* and *eGFP* in transgenic embryos shows colocalization (arrows) of the endogenous gene (magenta) and the enhancer reporter (GFP, *Axud1E1-SE*, turquoise) (**D’**). **(E-F)** Transverse sections of HH8 (**E**) and HH10 (**F**) embryos transfected with constructs *Axud1E1-500* (magenta) and *Axud1E1-SE* (*turquoise*). *Axud1E1-500* and *Axud1E1-SE* are expressed in the same pre-migratory (arrowheads in **D**) and migratory neural crest cells (arrowheads in **E**). **(G)** Quantification (RT-PCR) of eRNA depicting enrichment of *Axud1E1-500* in neural crest cells. While *Axud1E1* is actively transcribed, shadow element *Axud1E1-SE* is silent in dissected neural folds. Error bars represent ± SEM. Statistical significance determined via an unpaired t-test. HH, Hamburger and Hamilton. Scale bars represent 100μm (**A, B, D**) and 50μm (**E-F**). ***p < 0.001.
(TIF)

S3 Fig Tissue-specific expression of Wnt nuclear effector LEF1 in neural crest cells. **(A)** LEF1 colocalizes with the neural crest cell marker TFAP2B. Double immunohistochemistry (transverse section) for LEF1 and TFAP2B (**A**). **A’** and **A”** show higher magnification of the area indicated in **A**.
(TIF)

S4 Fig *Axud1E1* mutant variants are active in *AXUD1*+ cells. **(A-B)** Double fluorescent *in situ* hybridization for *AXUD1* and e*GFP* in transgenic embryos shows colocalization (arrows) of the endogenous gene (magenta) and the enhancer reporter (turquoise) for *Axud1E1-mutAB* (**A-A’**) and *Axud1E1-300* (**B-B’**). HH, Hamburger and Hamilton; Scale bars represent 100μm.
(TIF)

S5 Fig Additional analysis for loss-of function analysis of *Axud1E1* upstream regulators. **(A)** Western blot for SP5 and TFAP2A in embryos bilaterally transfected with SP5 and TFAP2A morpholinos, respectively. **(B)** Quantification of relative number of Caspase-3 positive cells at the dorsal neural tube in embryos transfected with WNT1/4, SP5 and TFAP2A morpholinos. Error bars represent ± SEM. The statistical significance was determined via an unpaired t-test. **(C)** Mutation of TFAP2A binding sites resulted in no change of *Axud1E1-300* activity. Embryos were transfected with wild-type enhancer (*Axud1E1-300*) on the left side and the TFAP2A mutant construct on the right side. **(D)** TFAP2A promotes *Axud1E1* accessibility. Quantification of chromatin accessibility (ATAC-qPCR) of embryos transfected with TFAP2A morpholino shows loss of *Axud1E1* accessibility following knockdown of the pioneer factor. The *FOXD3* enhancer (Foxd3NC1), which is not bound by TFAP2A, is unaffected by *TFAP2A* loss-of-function. Error bars represent ± SEM. The statistical significance was determined via unpaired t-test. HH, Hamburger and Hamilton; Scale bars represent 100μm (**C**); **p < 0.01.
(TIF)

S6 Fig *SP5* expression during neural crest development. **(A-C)** *SP5* is transiently expressed in the neural crest lineage. Whole mount *in situ* hybridization (**A-C**) and transverse sections (**A’-C’**) show expression of *SP5* in the neural plate border (**A** and **A’**) and dorsal neural folds (**B** and **B’**). During neural crest specification, *SP5* is excluded from the dorsal neural tube (**C** and arrow in **C’**). **(D)** *SP5* expression levels in the neural crest lineage. Data from RNA-seq analysis shows rapid decrease of *SP5* mRNA levels during neural crest development from stages HH6 to HH16. **(E-G)** *SP5* expression colocalizes with the neural crest markers *PAX7* (arrows in **E**) and *TFAP2B* (**F**) in early neural crest cells. Colocalization of *SP5* and *TFAP2B* is lost in migratory/late neural crest cells (**G**, arrows in **G’** and **G”**). **G’** and **G”** present a magnification of the area highlighted in **G**. HH, Hamburger and Hamilton; Scale bars represent 500μm (**A-C**), 100μm (**A’-C’**); 200μm (**E-G**).
(TIF)

S7 Fig SP5 directly regulates neural plate border genes *TFAP2A*, *MSX1* and *ZIC1*. **(A)** Chromatin immunoprecipitation for CTNNB1 shows temporal changes in the regulation of *SP5* and *AXUD1* by Wnt signaling. Association of CTNNB1 with an *SP5* enhancer (*Sp5E*) decreases during neural crest specification. Conversely, binding of the Wnt effector to *Axud1E1* significantly increases from HH5 to HH9. Error bars represent ± SEM. The statistical significance was determined via an unpaired t-test. **(B)** Fragment size distribution of the two replicates of SP5 CUT&RUN read pairs. Motif enrichment analysis via HOMER for regions occupied by SP5 shows enrichment for GC boxes, similar to other Sp/Klf Zn2+-finger transcription factors. pValue indicates significance of motif occurrence as reported by HOMER. **(C)** Pairwise Pearson correlation of SP5 and H3K27Ac CUT&RUN replicates. **(D)** CUT&RUN profiles of SP5 and H3K27Ac at the cis-regulatory elements *Tfap2aE1*, *Msx1E1* and *Zic1E1*. The three elements are robustly active in the neural crest lineage. HH, Hamburger and Hamilton; Scale bars represent 200μm (**D**); ***p < 0.001.
(TIF)

## References

[1] Le Douarin N, Kalcheim C. The neural crest. 2nd ed Cambridge: Cambridge University Press; 1999 xxiii, 445p. p.

[2] Meulemans D, Bronner-Fraser M. Gene-regulatory interactions in neural crest evolution and development. Developmental cell. 2004;7(3):291–9. 10.1016/j.devcel.2004.08.007 15363405

[3] Sauka-Spengler T, Bronner-Fraser M. A gene regulatory network orchestrates neural crest formation. Nature reviews Molecular cell biology. 2008;9(7):557–68. 10.1038/nrm2428 18523435

[4] Simoes-Costa M, Bronner ME. Establishing neural crest identity: a gene regulatory recipe. Development. 2015;142(2):242–57. 10.1242/dev.105445 25564621 PMC4302844

[5] Stuhlmiller TJ, Garcia-Castro MI. Current perspectives of the signaling pathways directing neural crest induction. Cellular and molecular life sciences: CMLS. 2012;69(22):3715–37. 10.1007/s00018-012-0991-8 22547091 PMC3478512

[6] Martik ML, Gandhi S, Uy BR, Gillis JA, Green SA, Simoes-Costa M, et al Evolution of the new head by gradual acquisition of neural crest regulatory circuits. Nature. 2019;574(7780):675–8. 10.1038/s41586-019-1691-4 31645763 PMC6858584

[7] Simoes-Costa M, Bronner ME. Reprogramming of avian neural crest axial identity and cell fate. Science. 2016;352(6293):1570–3. 10.1126/science.aaf2729 27339986 PMC5100669

[8] Bhattacharya D, Azambuja AP, Simoes Costa S. Metabolic reprogramming promotes neural crest migration via Yap/Tead signaling. Developmental cell. 2020;In press. 10.1016/j.devcel.2020.03.005 32243782 PMC7236757

[9] Davidson EH, Levine MS. Properties of developmental gene regulatory networks. Proc Natl Acad Sci U S A. 2008;105(51):20063–6. 10.1073/pnas.0806007105 19104053 PMC2629280

[10] Davidson EH, McClay DR, Hood L. Regulatory gene networks and the properties of the developmental process. Proceedings of the National Academy of Sciences of the United States of America. 2003;100(4):1475–80. 10.1073/pnas.0437746100 12578984 PMC149855

[11] Peter IS, Davidson EH. Assessing regulatory information in developmental gene regulatory networks. Proceedings of the National Academy of Sciences of the United States of America. 2017;114(23):5862–9. 10.1073/pnas.1610616114 28584110 PMC5468647

[12] Hovland AS, Rothstein M, Simoes-Costa M. Network architecture and regulatory logic in neural crest development. Wiley Interdiscip Rev Syst Biol Med. 2019:e1468 10.1002/wsbm.1468 31702881 PMC7236752

[13] Simoes-Costa M, Stone M, Bronner ME. Axud1 Integrates Wnt Signaling and Transcriptional Inputs to Drive Neural Crest Formation. Dev Cell. 2015;34(5):544–54. 10.1016/j.devcel.2015.06.024 26256212 PMC4573882

[14] Basch ML, Bronner-Fraser M, Garcia-Castro MI. Specification of the neural crest occurs during gastrulation and requires Pax7. Nature. 2006;441(7090):218–22. 10.1038/nature04684 16688176

[15] Roellig D, Tan-Cabugao J, Esaian S, Bronner ME. Dynamic transcriptional signature and cell fate analysis reveals plasticity of individual neural plate border cells. Elife. 2017;6 10.7554/eLife.21620 28355135 PMC5371430

[16] Garcia-Castro MI, Marcelle C, Bronner-Fraser M. Ectodermal Wnt Function As a Neural Crest Inducer. Science. 2002;13:13 12161657 10.1126/science.1070824

[17] Rothstein M, Simoes-Costa M. Heterodimerization of TFAP2 pioneer factors drives epigenomic remodeling during neural crest specification. Genome Res. 2020;30(1):35–48. 10.1101/gr.249680.119 31848212 PMC6961570

[18] Rada-Iglesias A, Bajpai R, Prescott S, Brugmann SA, Swigut T, Wysocka J. Epigenomic annotation of enhancers predicts transcriptional regulators of human neural crest. Cell stem cell. 2012;11(5):633–48. 10.1016/j.stem.2012.07.006 22981823 PMC3751405

[19] Uchikawa M, Ishida Y, Takemoto T, Kamachi Y, Kondoh H. Functional analysis of chicken Sox2 enhancers highlights an array of diverse regulatory elements that are conserved in mammals. Developmental cell. 2003;4(4):509–19. 10.1016/s1534-5807(03)00088-1 12689590

[20] Williams RM, Candido-Ferreira I, Repapi E, Gavriouchkina D, Senanayake U, Ling ITC, et al Reconstruction of the Global Neural Crest Gene Regulatory Network In Vivo. Developmental cell. 2019;51(2):255–76 e7. 10.1016/j.devcel.2019.10.003 31639368 PMC6838682

[21] Corish P, Tyler-Smith C. Attenuation of green fluorescent protein half-life in mammalian cells. Protein Eng. 1999;12(12):1035–40. 10.1093/protein/12.12.1035 10611396

[22] Hong JW, Hendrix DA, Levine MS. Shadow enhancers as a source of evolutionary novelty. Science. 2008;321(5894):1314 10.1126/science.1160631 18772429 PMC4257485

[23] Gandhi S, Piacentino ML, Vieceli FM, Bronner ME. Optimization of CRISPR/Cas9 genome editing for loss-of-function in the early chick embryo. Developmental biology. 2017;432(1):86–97. 10.1016/j.ydbio.2017.08.036 29150011 PMC5728388

[24] Simoes-Costa MS, McKeown SJ, Tan-Cabugao J, Sauka-Spengler T, Bronner ME. Dynamic and differential regulation of stem cell factor FoxD3 in the neural crest is Encrypted in the genome. PLoS genetics. 2012;8(12):e1003142 10.1371/journal.pgen.1003142 23284303 PMC3527204

[25] Betancur P, Bronner-Fraser M, Sauka-Spengler T. Genomic code for Sox10 activation reveals a key regulatory enhancer for cranial neural crest. Proceedings of the National Academy of Sciences of the United States of America. 2010;107(8):3570–5. 10.1073/pnas.0906596107 20139305 PMC2840498

[26] Fujimura N, Vacik T, Machon O, Vlcek C, Scalabrin S, Speth M, et al Wnt-mediated down-regulation of Sp1 target genes by a transcriptional repressor Sp5. The Journal of biological chemistry. 2007;282(2):1225–37. 10.1074/jbc.M605851200 17090534

[27] Skene PJ, Henikoff S. An efficient targeted nuclease strategy for high-resolution mapping of DNA binding sites. Elife. 2017;6 10.7554/eLife.21856 28079019 PMC5310842

[28] Harrison SM, Houzelstein D, Dunwoodie SL, Beddington RS. Sp5, a new member of the Sp1 family, is dynamically expressed during development and genetically interacts with Brachyury. Dev Biol. 2000;227(2):358–72. 11071760 10.1006/dbio.2000.9878

[29] Davidson E. The Regulatory Genome: Gene Regulatory Networks in Development and Evolution. Burlington, MA: Elsevier; 2009 10.1111/j.1558-5646.2009.00908.x

[30] Barembaum M, Bronner ME. Identification and dissection of a key enhancer mediating cranial neural crest specific expression of transcription factor, Ets-1. Developmental biology. 2013 10.1016/j.ydbio.2013.08.009 23969311 PMC3872135

[31] Vadasz S, Marquez J, Tulloch M, Shylo NA, Garcia-Castro MI. Pax7 is regulated by cMyb during early neural crest development through a novel enhancer. Development. 2013;140(17):3691–702. 10.1242/dev.088328 23942518 PMC3742149

[32] Antonellis A, Huynh JL, Lee-Lin SQ, Vinton RM, Renaud G, Loftus SK, et al Identification of neural crest and glial enhancers at the mouse Sox10 locus through transgenesis in zebrafish. PLoS genetics. 2008;4(9):e1000174 10.1371/journal.pgen.1000174 18773071 PMC2518861

[33] Simoes-Costa M, Tan-Cabugao J, Antoshechkin I, Sauka-Spengler T, Bronner ME. Transcriptome analysis reveals novel players in the cranial neural crest gene regulatory network. Genome Res. 2014;24(2):281–90. 10.1101/gr.161182.113 24389048 PMC3912418

[34] Tani-Matsuhana S, Vieceli FM, Gandhi S, Inoue K, Bronner ME. Transcriptome profiling of the cardiac neural crest reveals a critical role for MafB. Developmental biology. 2018;444 Suppl 1:S209–S18. 10.1016/j.ydbio.2018.09.015 30236445 PMC6421117

[35] Alon U. Network motifs: theory and experimental approaches. Nature reviews Genetics. 2007;8(6):450–61. 10.1038/nrg2102 17510665

[36] Huggins IJ, Bos T, Gaylord O, Jessen C, Lonquich B, Puranen A, et al The WNT target SP5 negatively regulates WNT transcriptional programs in human pluripotent stem cells. Nature communications. 2017;8(1):1034 10.1038/s41467-017-01203-1 29044119 PMC5647328

[37] Park DS, Seo JH, Hong M, Bang W, Han JK, Choi SC. Role of Sp5 as an essential early regulator of neural crest specification in xenopus. Developmental dynamics: an official publication of the American Association of Anatomists. 2013;242(12):1382–94. 10.1002/dvdy.24034 24038420

[38] Kennedy MW, Chalamalasetty RB, Thomas S, Garriock RJ, Jailwala P, Yamaguchi TP. Sp5 and Sp8 recruit beta-catenin and Tcf1-Lef1 to select enhancers to activate Wnt target gene transcription. Proceedings of the National Academy of Sciences of the United States of America. 2016;113(13):3545–50. 10.1073/pnas.1519994113 26969725 PMC4822596

[39] Bhattacharya D, Rothstein M, Azambuja AP, Simoes-Costa M. Control of neural crest multipotency by Wnt signaling and Lin28/let-7 axis. Under review. 2018 10.7554/eLife.40556 30520734 PMC6301792

[40] Taneyhill LA, Coles EG, Bronner-Fraser M. Snail2 directly represses cadherin6B during epithelial-tomesenchymal transitions of the neural crest. Development. 2007;134(8):1481–90. 10.1242/dev.02834 17344227 PMC2595139

[41] Kutejova E, Sasai N, Shah A, Gouti M, Briscoe J. Neural Progenitors Adopt Specific Identities by Directly Repressing All Alternative Progenitor Transcriptional Programs. Developmental cell. 2016;36(6):639–53. 10.1016/j.devcel.2016.02.013 26972603 PMC4819439

[42] Lukoseviciute M, Gavriouchkina D, Williams RM, Hochgreb-Hagele T, Senanayake U, Chong-Morrison V, et al From Pioneer to Repressor: Bimodal foxd3 Activity Dynamically Remodels Neural Crest Regulatory Landscape In Vivo. Developmental cell. 2018;47(5):608–28 e6. 10.1016/j.devcel.2018.11.009 30513303 PMC6286384

[43] Chapman SC, Collignon J, Schoenwolf GC, Lumsden A. Improved method for chick whole-embryo culture using a filter paper carrier. Developmental dynamics: an official publication of the American Association of Anatomists. 2001;220(3):284–9. 10.1002/1097-0177(20010301)220:3&lt;284::AID-DVDY1102&gt;3.0.CO;2-5 11241836

[44] Hamburger V, Hamilton HL. A series of normal stages in the development of the chick embryo. Journal of morphology. 1951;88(1):49–92. 24539719

[45] Wilkinson DG. In situ hybridization: a practical approach. Oxford; New York: IRL Press at Oxford University Press; 1992 xvii, 163 p. p.

[46] Denkers N, Garcia-Villalba P, Rodesch CK, Nielson KR, Mauch TJ. FISHing for chick genes: Triple-label whole-mount fluorescence in situ hybridization detects simultaneous and overlapping gene expression in avian embryos. Dev Dyn. 2004;229(3):651–7. 10.1002/dvdy.20005 14991720

[47] Naumova N, Smith EM, Zhan Y, Dekker J. Analysis of long-range chromatin interactions using Chromosome Conformation Capture. Methods. 2012;58(3):192–203. 10.1016/j.ymeth.2012.07.022 22903059 PMC3874837

[48] Stadhouders R, Kolovos P, Brouwer R, Zuin J, van den Heuvel A, Kockx C, et al Multiplexed chromosome conformation capture sequencing for rapid genome-scale high-resolution detection of long-range chromatin interactions. Nat Protoc. 2013;8(3):509–24. 10.1038/nprot.2013.018 23411633

[49] Hagege H, Klous P, Braem C, Splinter E, Dekker J, Cathala G, et al Quantitative analysis of chromosome conformation capture assays (3C-qPCR). Nature protocols. 2007;2(7):1722–33. 10.1038/nprot.2007.243 17641637

[50] Ea V, Court F, Forne T. Quantitative Analysis of Intra-chromosomal Contacts: The 3C-qPCR Method. Methods Mol Biol. 2017;1589:75–88. 10.1007/7651_2015_269 26025624

[51] Mathelier A, Fornes O, Arenillas DJ, Chen CY, Denay G, Lee J, et al JASPAR 2016: a major expansion and update of the open-access database of transcription factor binding profiles. Nucleic acids research. 2016;44(D1):D110–5. 10.1093/nar/gkv1176 26531826 PMC4702842

[52] Betancur P, Simoes-Costa M, Sauka-Spengler T, Bronner ME. Expression and function of transcription factor cMyb during cranial neural crest development. Mech Dev. 2014;132:38–43. 10.1016/j.mod.2014.01.005 24509349 PMC3987950

[53] Martin M. Cutadapt removes adapter sequences from high-throughput sequencing reads. EMBnetjournal. 2010;17:10–2.

[54] Langmead B, Salzberg SL. Fast gapped-read alignment with Bowtie 2. Nat Methods. 2012;9(4):357–9. 10.1038/nmeth.1923 22388286 PMC3322381

[55] Ramirez F, Ryan DP, Gruning B, Bhardwaj V, Kilpert F, Richter AS, et al deepTools2: a next generation web server for deep-sequencing data analysis. Nucleic Acids Res. 2016;44(W1):W160–5. 10.1093/nar/gkw257 27079975 PMC4987876

[56] Yu G, Wang LG, Han Y, He QY. clusterProfiler: an R package for comparing biological themes among gene clusters. OMICS. 2012;16(5):284–7. 10.1089/omi.2011.0118 22455463 PMC3339379

[57] Heinz S, Benner C, Spann N, Bertolino E, Lin YC, Laslo P, et al Simple combinations of lineage-determining transcription factors prime cis-regulatory elements required for macrophage and B cell identities. Molecular cell. 2010;38(4):576–89. 10.1016/j.molcel.2010.05.004 20513432 PMC2898526

