# Supplementary figures and images for: A regulatory sub-circuit downstream of Wnt signaling controls developmental transitions in neural crest formation
Source: PLoS Genet. 2021 Jan 19;17(1):e1009296. doi: 10.1371/journal.pgen.1009296 (PMC7846109; doi:10.1371/journal.pgen.1009296)

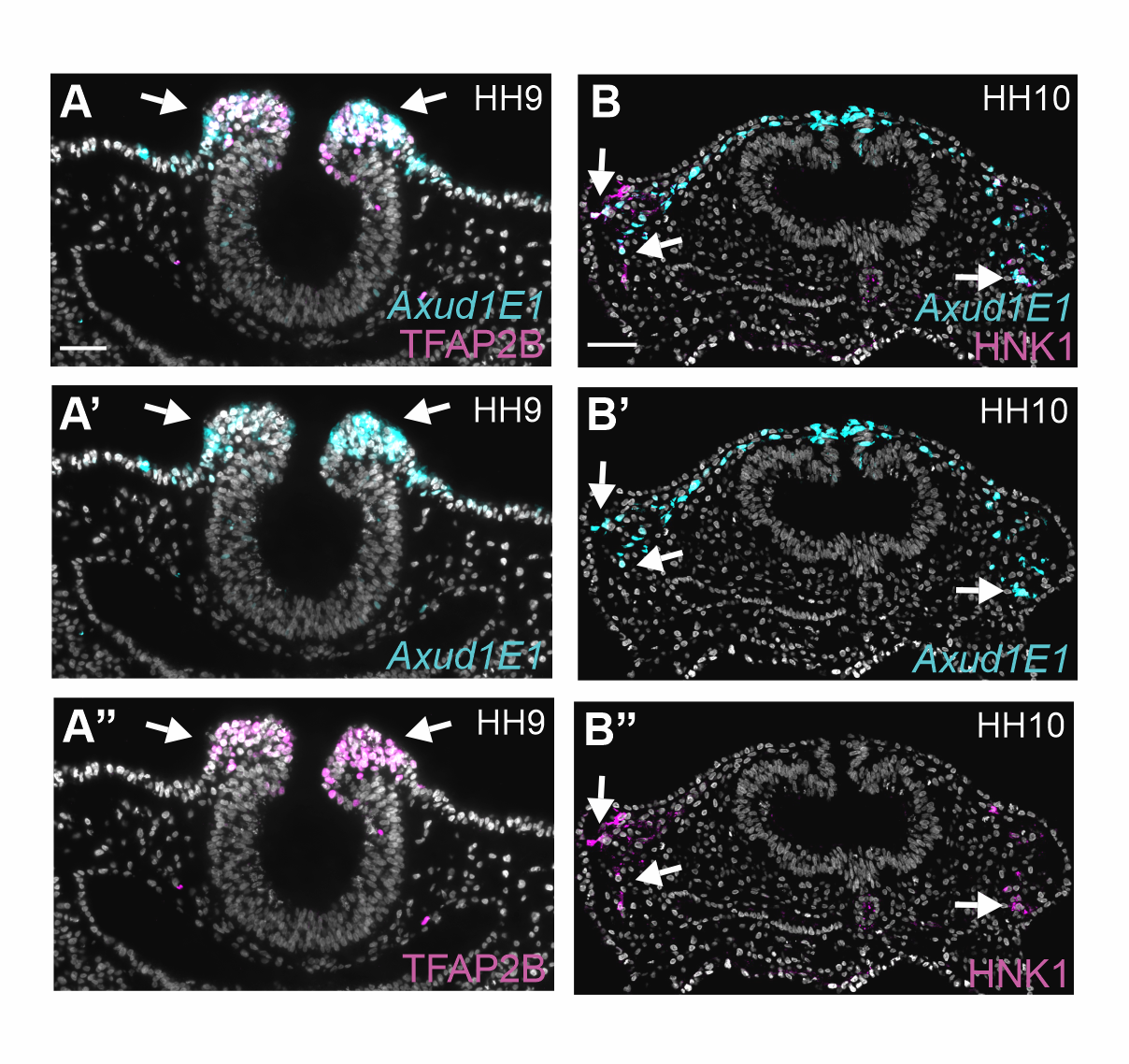

Supplement: S1 Fig — (A) Immunohistochemistry for the endogenous TFAP2B protein (magenta) in transverse sections of an HH9 transgenic embryo transfected with Axud1E1:eGFP (turquoise) (A). Reporter expression indicates enhancer activity (A’) in TFAP2B+ cells located at the dorsal neural folds (A”). (B) Immunohistochemistry (transverse sections) for HNK1 marker (magenta) in an HH10 transgenic embryo transfected with Axud1E1:eGFP (turquoise) (B). Arrows indicate specific activity of Axud1E1 (B’) in migratory HNK1+ cells (B”). HH, Hamburger and Hamilton; Scale bars represent 50μm. (TIF) [file pgen.1009296.s006.tif]

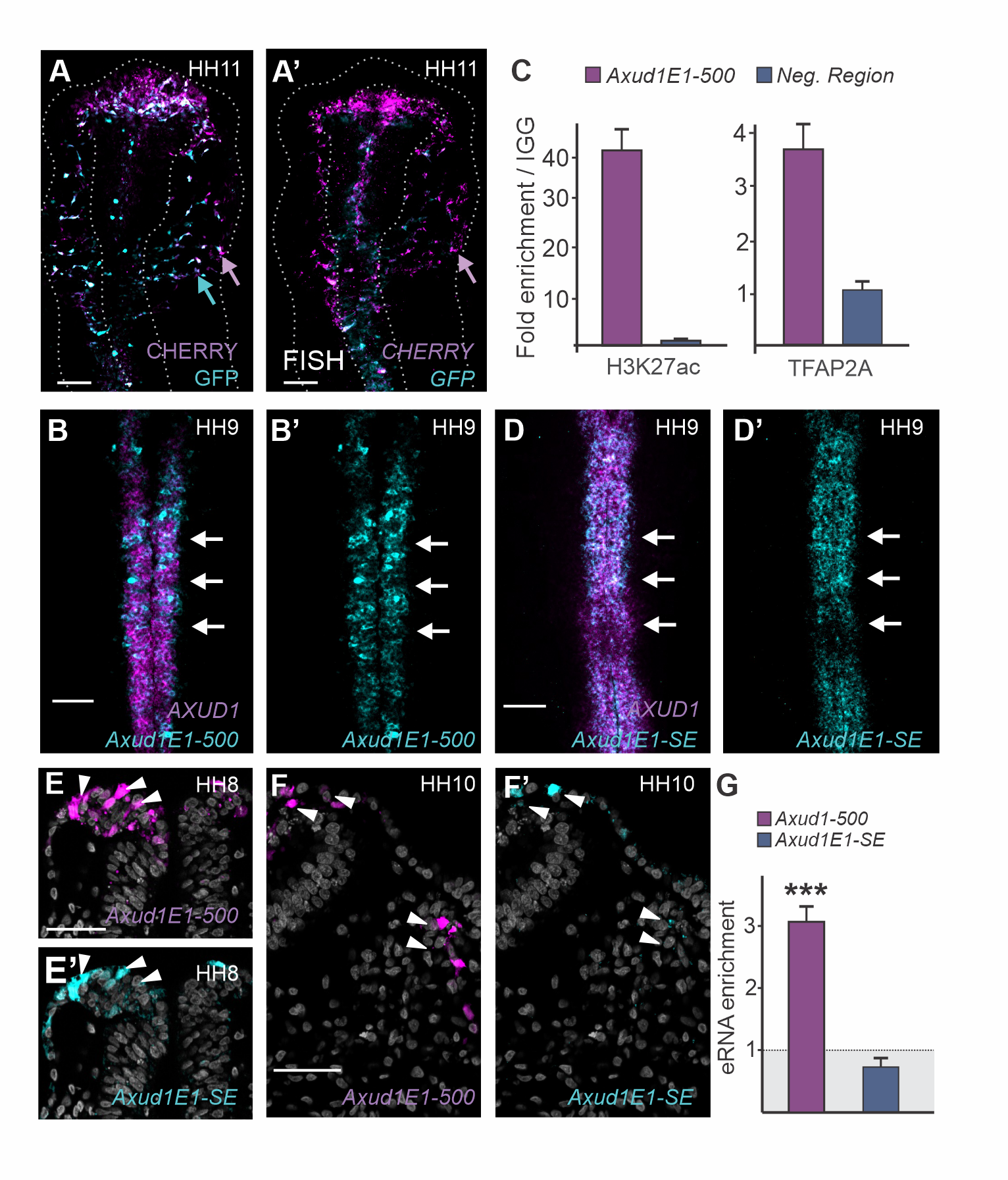

Supplement: S2 Fig — (A-A’) Extended Axud1E1 reporter activity in late migratory neural crest cells is due to GFP stability. (A) Transient transgenesis expression pattern of Tfap2aE1:mChe (magenta) and Axud1E1:GFP depicting Axud1E1 reporter activity in late migratory neural crest cells (arrows). (A’) Same embryo presented in (A) after double fluorescent in situ hybridization targeting mcherry and gfp indicated reduced expression of Axud1E1 in late migratory cells (arrows). (B) Double fluorescent in situ hybridization for AXUD1 and eGFP in transgenic embryos shows colocalization (arrows) of the endogenous gene (magenta) and the enhancer reporter (GFP, Axud1E1-500, turquoise) (B’). (C) Representative ChIP-qPCR experiment for the active chromatin mark H3K27ac and the neural crest pioneer factor TFAP2A indicates that Axud1E1 is an active neural crest enhancer. (D) Double fluorescent in situ hybridization for AXUD1 and eGFP in transgenic embryos shows colocalization (arrows) of the endogenous gene (magenta) and the enhancer reporter (GFP, Axud1E1-SE, turquoise) (D’). (E-F) Transverse sections of HH8 (E) and HH10 (F) embryos transfected with constructs Axud1E1-500 (magenta) and Axud1E1-SE (turquoise). Axud1E1-500 and Axud1E1-SE are expressed in the same pre-migratory (arrowheads in D) and migratory neural crest cells (arrowheads in E). (G) Quantification (RT-PCR) of eRNA depicting enrichment of Axud1E1-500 in neural crest cells. While Axud1E1 is actively transcribed, shadow element Axud1E1-SE is silent in dissected neural folds. Error bars represent ± SEM. Statistical significance determined via an unpaired t-test. HH, Hamburger and Hamilton. Scale bars represent 100μm (A, B, D) and 50μm (E-F). ***p < 0.001. (TIF) [file pgen.1009296.s007.tif]

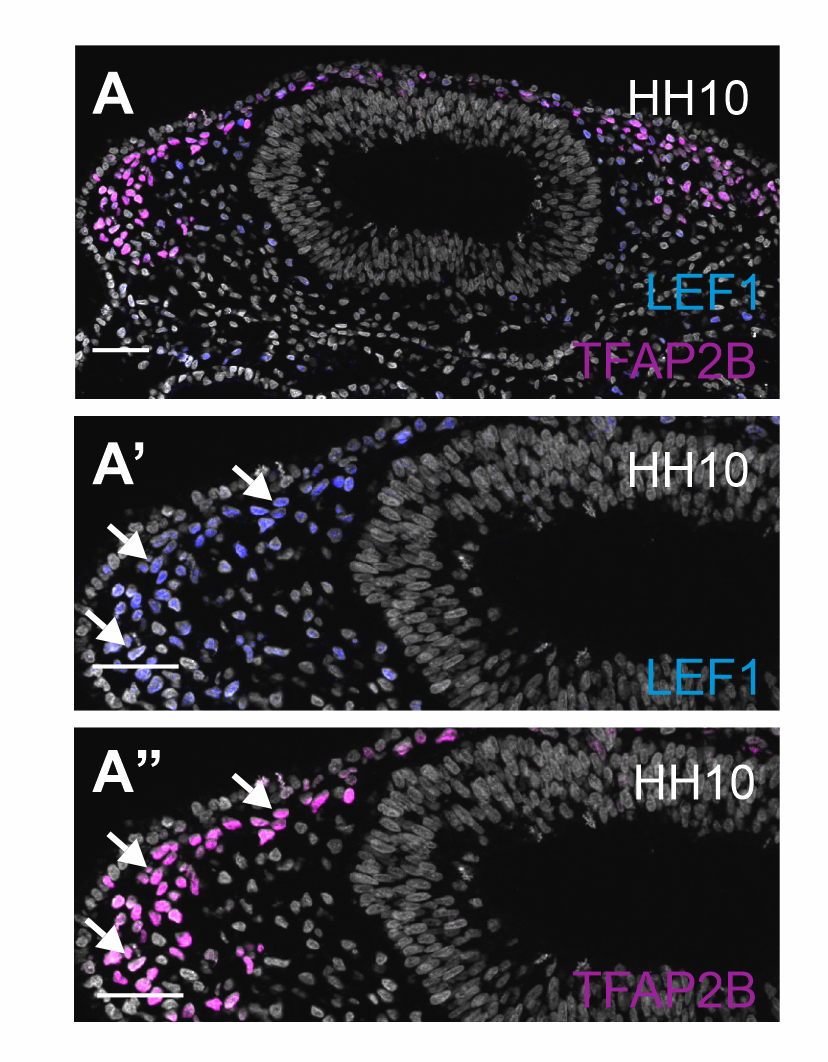

Supplement: S3 Fig — (A) LEF1 colocalizes with the neural crest cell marker TFAP2B. Double immunohistochemistry (transverse section) for LEF1 and TFAP2B (A). A’ and A” show higher magnification of the area indicated in A. (TIF) [file pgen.1009296.s008.tif]

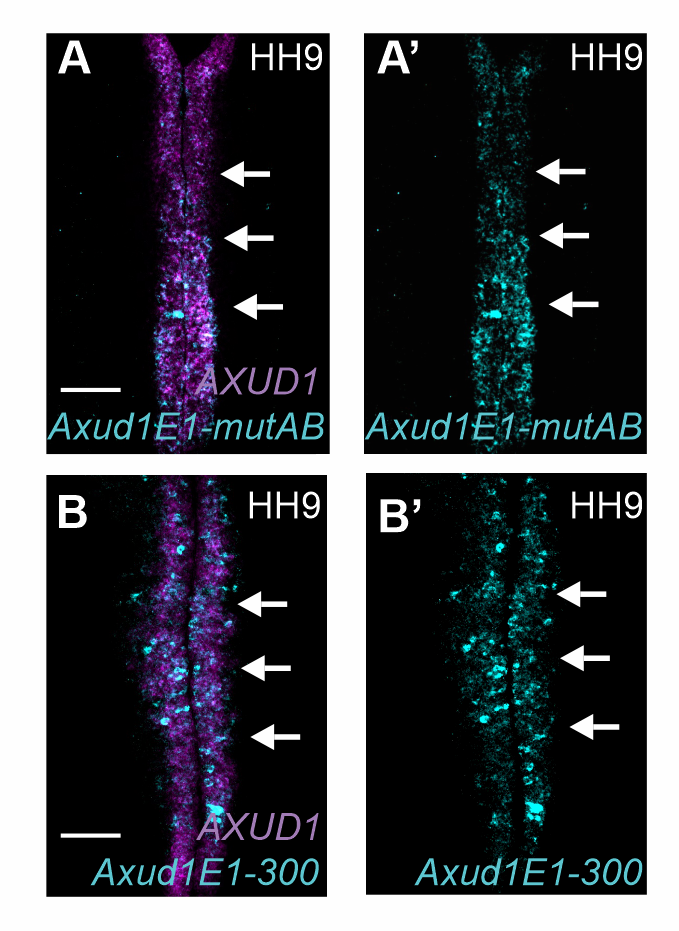

Supplement: S4 Fig — (A-B) Double fluorescent in situ hybridization for AXUD1 and eGFP in transgenic embryos shows colocalization (arrows) of the endogenous gene (magenta) and the enhancer reporter (turquoise) for Axud1E1-mutAB (A-A’) and Axud1E1-300 (B-B’). HH, Hamburger and Hamilton; Scale bars represent 100μm. (TIF) [file pgen.1009296.s009.tif]

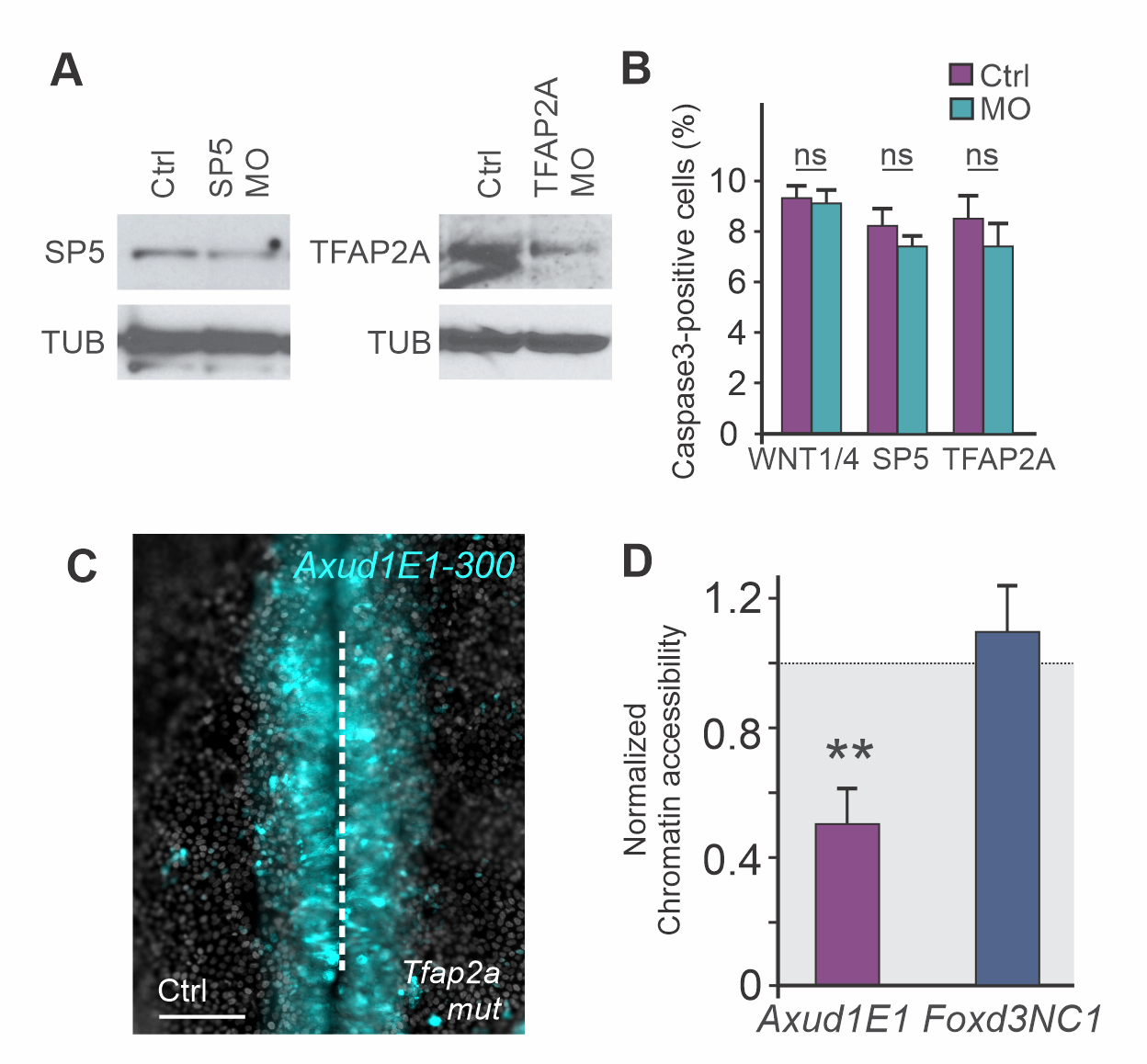

Supplement: S5 Fig — (A) Western blot for SP5 and TFAP2A in embryos bilaterally transfected with SP5 and TFAP2A morpholinos, respectively. (B) Quantification of relative number of Caspase-3 positive cells at the dorsal neural tube in embryos transfected with WNT1/4, SP5 and TFAP2A morpholinos. Error bars represent ± SEM. The statistical significance was determined via an unpaired t-test. (C) Mutation of TFAP2A binding sites resulted in no change of Axud1E1-300 activity. Embryos were transfected with wild-type enhancer (Axud1E1-300) on the left side and the TFAP2A mutant construct on the right side. (D) TFAP2A promotes Axud1E1 accessibility. Quantification of chromatin accessibility (ATAC-qPCR) of embryos transfected with TFAP2A morpholino shows loss of Axud1E1 accessibility following knockdown of the pioneer factor. The FOXD3 enhancer (Foxd3NC1), which is not bound by TFAP2A, is unaffected by TFAP2A loss-of-function. Error bars represent ± SEM. The statistical significance was determined via unpaired t-test. HH, Hamburger and Hamilton; Scale bars represent 100μm (C); **p < 0.01. (TIF) [file pgen.1009296.s010.tif]

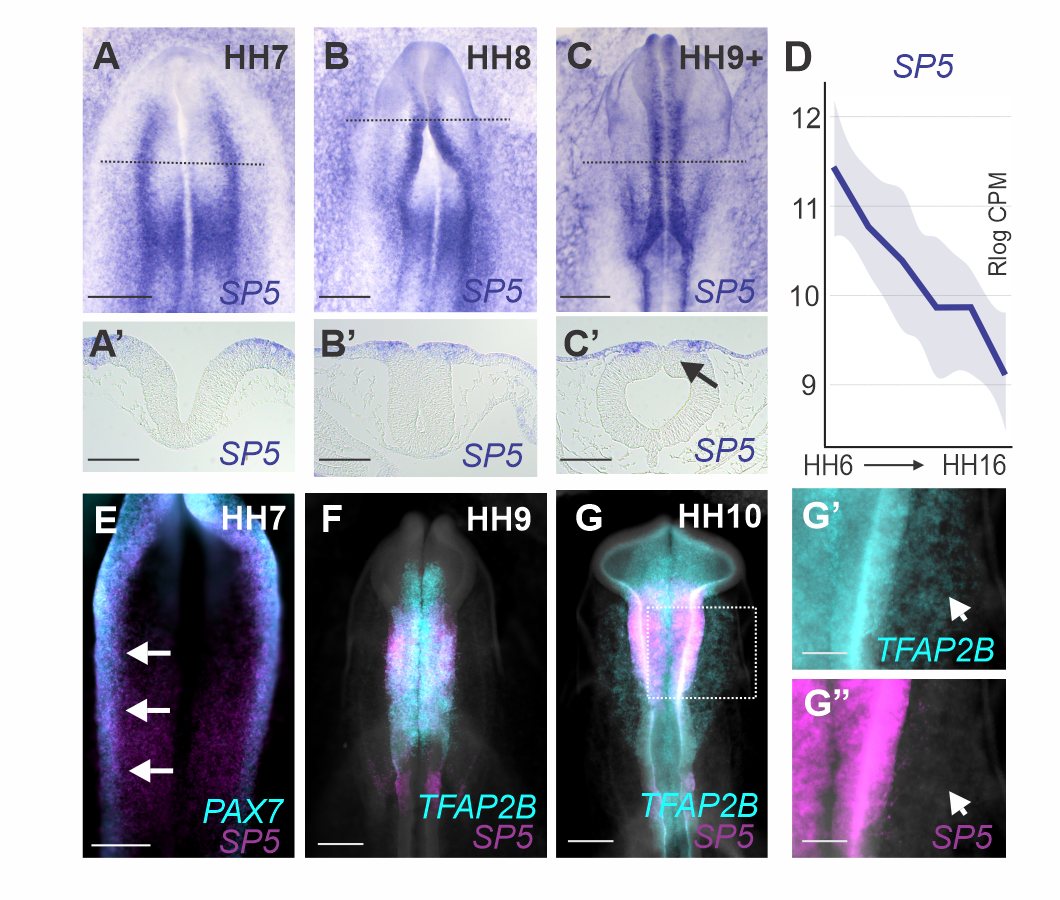

Supplement: S6 Fig — (A-C) SP5 is transiently expressed in the neural crest lineage. Whole mount in situ hybridization (A-C) and transverse sections (A’-C’) show expression of SP5 in the neural plate border (A and A’) and dorsal neural folds (B and B’). During neural crest specification, SP5 is excluded from the dorsal neural tube (C and arrow in C’). (D) SP5 expression levels in the neural crest lineage. Data from RNA-seq analysis shows rapid decrease of SP5 mRNA levels during neural crest development from stages HH6 to HH16. (E-G) SP5 expression colocalizes with the neural crest markers PAX7 (arrows in E) and TFAP2B (F) in early neural crest cells. Colocalization of SP5 and TFAP2B is lost in migratory/late neural crest cells (G, arrows in G’ and G”). G’ and G” present a magnification of the area highlighted in G. HH, Hamburger and Hamilton; Scale bars represent 500μm (A-C), 100μm (A’-C’); 200μm (E-G). (TIF) [file pgen.1009296.s011.tif]

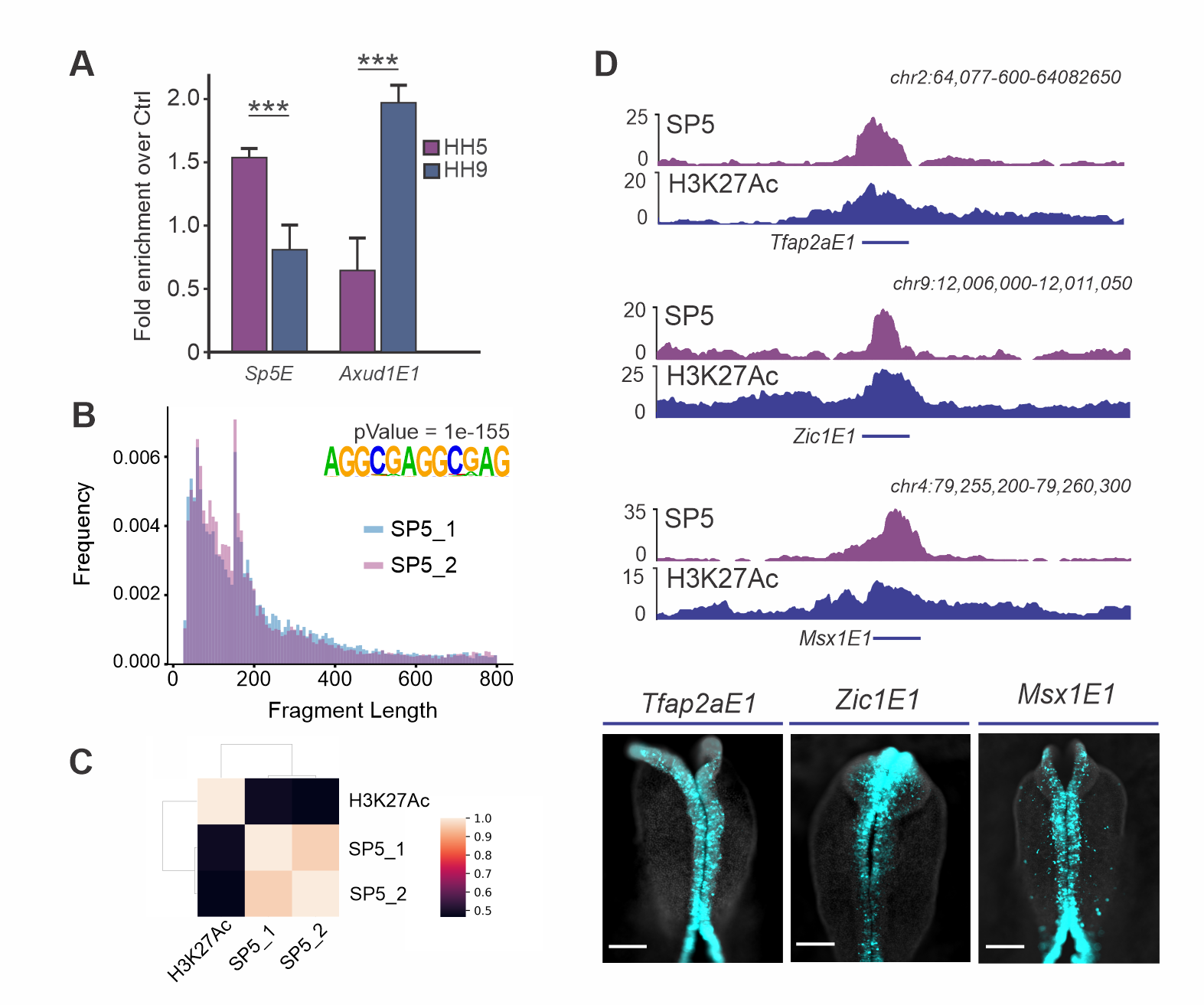

Supplement: S7 Fig — (A) Chromatin immunoprecipitation for CTNNB1 shows temporal changes in the regulation of SP5 and AXUD1 by Wnt signaling. Association of CTNNB1 with an SP5 enhancer (Sp5E) decreases during neural crest specification. Conversely, binding of the Wnt effector to Axud1E1 significantly increases from HH5 to HH9. Error bars represent ± SEM. The statistical significance was determined via an unpaired t-test. (B) Fragment size distribution of the two replicates of SP5 CUT&RUN read pairs. Motif enrichment analysis via HOMER for regions occupied by SP5 shows enrichment for GC boxes, similar to other Sp/Klf Zn2+-finger transcription factors. pValue indicates significance of motif occurrence as reported by HOMER. (C) Pairwise Pearson correlation of SP5 and H3K27Ac CUT&RUN replicates. (D) CUT&RUN profiles of SP5 and H3K27Ac at the cis-regulatory elements Tfap2aE1, Msx1E1 and Zic1E1. The three elements are robustly active in the neural crest lineage. HH, Hamburger and Hamilton; Scale bars represent 200μm (D); ***p < 0.001. (TIF) [file pgen.1009296.s012.tif]
